# T-cell subset biomarkers across the rheumatoid arthritis disease continuum: from clinical utility to adoption in daily practice

**DOI:** 10.1093/rheumatology/keag286

**Published:** 2026-06-10

**Authors:** Innocent C Anioke, Fatih Tastekin, Gulay Alp, Helen Ng, Preveena Ravi, Isobel Parker, Min Lou, Hanna Gul, Laurence Duquenne, Edith Villeneuve, Jacqueline L Nam, Sana Sharrack, Maya H Buch, Philip G Conaghan, Kulveer Mankia, Paul Emery, Frederique Ponchel

**Affiliations:** Leeds Institute of Rheumatic and Musculoskeletal Medicine, The University of Leeds, Leeds, UK; Leeds Institute of Rheumatic and Musculoskeletal Medicine, The University of Leeds, Leeds, UK; Leeds Institute of Rheumatic and Musculoskeletal Medicine, The University of Leeds, Leeds, UK; Leeds Institute of Rheumatic and Musculoskeletal Medicine, The University of Leeds, Leeds, UK; Leeds Institute of Rheumatic and Musculoskeletal Medicine, The University of Leeds, Leeds, UK; Leeds Institute of Rheumatic and Musculoskeletal Medicine, The University of Leeds, Leeds, UK; Leeds Institute of Rheumatic and Musculoskeletal Medicine, The University of Leeds, Leeds, UK; Leeds Institute of Rheumatic and Musculoskeletal Medicine, The University of Leeds, Leeds, UK; Leeds Institute of Rheumatic and Musculoskeletal Medicine, The University of Leeds, Leeds, UK; Leeds Institute of Rheumatic and Musculoskeletal Medicine, The University of Leeds, Leeds, UK; Leeds Teaching Hospitals NHS Trust, Leeds, UK; Leeds Institute of Rheumatic and Musculoskeletal Medicine, The University of Leeds, Leeds, UK; NIHR Leeds Biomedical Research Centre, Chapel Allerton Hospital, Leeds, UK; Leeds Institute of Rheumatic and Musculoskeletal Medicine, The University of Leeds, Leeds, UK; Division of Musculoskeletal & Dermatological Science, University of Manchester, Manchester, UK; Leeds Institute of Rheumatic and Musculoskeletal Medicine, The University of Leeds, Leeds, UK; NIHR Leeds Biomedical Research Centre, Chapel Allerton Hospital, Leeds, UK; Leeds Institute of Rheumatic and Musculoskeletal Medicine, The University of Leeds, Leeds, UK; Leeds Institute of Rheumatic and Musculoskeletal Medicine, The University of Leeds, Leeds, UK; NIHR Leeds Biomedical Research Centre, Chapel Allerton Hospital, Leeds, UK; Leeds Institute of Rheumatic and Musculoskeletal Medicine, The University of Leeds, Leeds, UK; Département de Rhumatologie, Université Jean Monnet Saint-Etienne, CHU Saint-Etienne, Mines Saint-Etienne, INSERM, SAINBIOSE U1059, Saint-Etienne, France

**Keywords:** CD4+ T-cell subsets, rheumatoid arthritis, first-line treatment, remission

## Abstract

**Objective:**

The biomarker potential of CD4+ T-cell subsets [naive, regulatory (Treg), inflammation-related cells (IRC)] in patients with rheumatoid arthritis (RA) has been described.

This article investigates the dynamic changes in these biomarkers across the RA disease continuum from at-risk to drug-induced remission.

**Methods:**

T-cell subset biomarker data were acquired using flow cytometry. Multiple group comparisons were performed using ANOVA test (with Bonferroni correction).

**Results:**

In individuals at risk of RA, longitudinal analysis showed that IRC frequencies increased just prior to onset of clinical synovitis, while naive T-cell frequencies reduced in those progressing to clinical synovitis, but increased in non-progressors. The use of naive/IRC data improved the accuracy of RA classification, especially in ACPA-negative patients. A distinct T-cell biomarker signature was observed in late-onset RA (>60 years old *vs* <59). In untreated RA, the predictive value of naive T-cell frequencies for methotrexate response was confirmed. For patients on methotrexate, naive T cells increased only between 6 and 12 months and only when in remission. IRC and Treg showed no consistent change. In patients treated with TNF inhibitors, naive T-cell frequency increased independently of response, whilst sustained IRC reductions and Treg increases were seen in remission. Once in stable clinical remission, only naive frequencies increased with the length of remission on conventional synthetic disease-modifying anti-rheumatic drugs (csDMARDs), whilst only Treg increased over time in TNF inhibitor-induced remission.

**Conclusion:**

These studies validated that T-cell subset measurements are independent from other currently used biomarkers, highlighting differences in the impact of drug modes of action on the three T-cell subsets. There is still an unmet need for biomarkers to predict response to TNF inhibition in early RA.

Rheumatology key messages

- T-cell subsets have predictive value for each outcome of the different stages of the inflammatory arthritis continuum.
- Confounding factors such as concomitant osteoarthritis and older age needs to be considered when using these biomarkers.
- These biomarkers can be obtained from hospital services, allowing easy implementation.

## Introduction

Rheumatoid arthritis (RA) is a chronic inflammatory disease that primarily affects synovial joints and causes damage to bone and cartilage, leading to pain, swelling and potential lifelong disability if untreated. The diagnosis relies on clinical signs, symptoms and autoantibodies [1], although these are not present in all patients at presentation, highlighting early disease heterogeneity. A pre-clinical RA phase also occurs [2, 3], characterized by systemic autoimmunity [presence of anti-citrullinated peptide antibodies (ACPA)] without clinical synovitis [4]. Given the destructive nature of RA, early remission is key to its management [5]. Thus, novel biomarkers are crucial for predicting progression in at-risk individuals and managing disease progression in people with RA in the short and long term.

In early, drug-naive RA, our group previously identified changes in the frequencies of CD4+ naive T cells and regulatory T cells (Treg). We also identified a sub-population of naive T cells which correlated with the C-reactive protein (CRP) that we called inflammation-related cells (IRC) [6, 7]. We further reported on the potential of these 3 CD4+ T-cell subsets as novel biomarkers for the management of RA patients.

In the pre-clinical phase of RA, we observed disturbances in naive, Treg and IRC frequencies [8]. Around 40% of at-risk cases progressed to undifferentiated and/or rheumatoid arthritis (UA/RA). Various prediction models using clinical and imaging data have been developed to predict progression [9–13]. Individual T-cell biomarkers were also predictive of progression to inflammatory arthritis, and their value when combined with clinical data was established in predictive models in 150 individuals [14]. After the onset of clinical synovitis, naive cells had potential for predicting RA classification in patients from an early arthritis clinic (EAC) [14]. A regression analysis suggested that only three variables [naive T cells, disease activity score (DAS) and age] contributed significantly to the model and achieved 80% accuracy.

Following RA classification, treatment initiation typically involves csDMARDs, most commonly methotrexate (MTX). Naive T-cell frequency was independently associated with achieving remission on MTX in early RA [15] and a predictive model was developed, selecting the three most predictive biomarkers (naive T cells, smoking status, DAS28 scores) in 70 patients [14]. Clinical trials have suggested the superiority of anti-tumour necrosis factor agents (TNFi) in achieving better long-term outcomes in early RA [16], but it remains unclear whether T-cell biomarkers can predict response to biological-DMARDs (bDMARDs), although data from nine patients suggested no association with outcome [15]. T-cell biomarkers were widely distributed in RA patients in clinical remission on either csDMARDs or bDMARDs, despite low disease activity (DAS28 < 2.6) [17]. The ability to sustain remission over 12 months once achieved on csDMARDs was associated with higher naive cells [14]. Successful tapering of csDMARDs was predicted by IRC in a model using ultrasound power Doppler and the Rheumatoid Arthritis Quality of Life questionnaire (RAQoL) [18]. Treg predicted flare in patients discontinuing a bDMARDs [19] and the successful tapering of bDMARDs was also predicted by Treg and IRC with CRP [20].

Following these initial reports, understanding the dynamic changes in these biomarkers across the early stages of RA and establishing whether they are impacted by demographic or clinical correlates, notably ACPA positivity, remains to be established. Here, data from 1727 participants across >2500 visits at distinct stages of the RA disease continuum are presented. While validating our prior work (detailed in the supplementary materials), our main goal was to understand these biomarkers’ dynamics and their relationships with other current RA biomarkers across the RA disease continuum, from the at-risk stage to drug-induced remission.

## Methods

### Patients

Ethical approval was obtained from National Research Ethics Committees at different phases of the RA continuum (National Research Ethics Service, West Yorkshire Ethics Committee: REC09/H1307/98, REC10/H1307/138, NCT02433184, REC06/Q1205/169). All participants provided informed consent prior to recruitment. Participants included in this analysis were selected based on availability of T cells subset data. Healthy controls (*n* = 174) were used to establish age-associations for naive and Treg measurements as previously described [14, 21]. Groups included ACPA+ at risk of RA, a group previously extensively described [8, 9], EAC patients evolving to RA/alternative classifications over time, early RA treated with MTX or with MTX+TNFi from the drug arm of two clinical trials [22, 23], and patients achieving DAS28 <2.6 remission. These groups are described in more detail in the supplementary materials.

### Clinical data

Standard data were recorded, including (although not for all groups) age, sex, autoantibody status (ACPA/RF), smoking (ever/never), duration of symptoms, disease activity or remission, HLA-DR status, early morning stiffness (EMS), tender joint count (78 joints), swollen/tender joint count (28 joints), C-reactive protein (CRP), erythrocyte sedimentation rate (ESR) and global health (GH). DAS28 was calculated where relevant.

### Cell staining and flow cytometry strategies

Subset quantification for the three subsets was performed by NHS hospital services, according to standardized protocols as previously described [14] between 2013 and 2020, using fresh blood. Gating strategies were previously described [14]. Subset frequencies were reported as % of CD4+ T cells and normalized as previously described for naive and Treg [14]. A reminder of the overall methodology is provided in the supplementary materials. Treg measurements were affected by the shortage of one antibody for several months.

To produce this data, raw data files were retrieved from NHS servers and analysis was performed by an experienced scientist (ICA). Issues were encountered, stemming from human or technical errors, or compromised blood quality. A detailed analysis of the acceptability of data leading to recommendations for implementing such biomarkers in daily practice is presented in the supplementary materials.

### Statistical analysis

Continuous variables were described using median, IQR and range. Nominal variables were described as *n* (%). Non-parametric tests were used throughout. For univariate analysis, continuous variable measures were compared between outcomes using Mann–Whitney *U* (MWU) tests and nominal variables with Chi^2^. Correlations were investigated with Spearman tests and described using rho-values. Multiple group analyses were performed using ANOVA with post-hoc 2-group analysis (MWU), using Bonferroni corrections. Analyses were conducted using SPSS 29. Significance for *P*-value was set at <0.05.

Multivariate analysis for prediction models is described in supplementary materials. Predictions were described with odds ratio (OR) and area under the curve (AUC). To evaluate the robustness of our predictive models, we used internal validation using a bootstrapping procedure (500 permutations) to correct for optimism in model performance (generating 95% CI AUC). All study cohorts contained missing data. The pattern of missingness was consistent with data missing at random (Little’s MCAR test *P < *0.0001). Regression-based imputation generated unbiased parameter estimates defining appropriate boundaries for each variable and the ‘Random Number Generators’ function, followed by the ‘Impute Missing Data Values’ (five iterations, in SPSS). Model estimates were combined (‘pooling’ function) to generate overall multiple-imputation estimates. The pooled dataset was compared with the original dataset (odds ratios remained stable) and data distribution checks confirmed no significant differences from non-imputed data. The pooled dataset was used for modelling.

## Results

Participants (*n* = 1727) at various stages of the RA continuum are described in Table 1 and Supplementary Fig. S1.

**Table 1:** Demographic and clinical data for all participating groups.

Cohorts/Variables	HC	Arthralgia	Arthralgia Onset of IA	Early RA	First MTX treatment	First Anti-TNF treatment	Remission synthetic	Remission biologic	UA/others	PsA
*n*	174	442	77	360	221	78	418	265	82	44
^$^Age (years)	43	51		51	57	49	59	56	48	45
	(24)	(18)		(19)	(17)	(21)	(19)	(22)	(26)	(18)
	[19–71]	[18–82]		[21–87]	[19–87	[25–80]	[19–87]	[22–85	[23–83]	[19–66]
Sex M	(41)	130 (29.6)		101 (28.0	70 (31.7)	25 (32.5)	150 (36.4)	82 (31.1)	25 (29.6)	18 (41.9)
F	(58)	311 (70.4)		259 (72.0)	151 (68.3)	53 (67.5)	268 (63.6)	182 (68.9)	57 (70.4)	26 (58.1)
^$^Duration of symptom/disease^b^		64		24	24	19.2	48	74	23	30
	NA	(124)		(25)	(27)	(16)	(38)	(119)	(34)	(67)
		[0–1830]		[1–104]	[2–105]	[3–80]	[1–260]	[3–430]	[6–72]	[8–176]
^$^Duration of remission (months)							11	9		
	NA	NA	NA	NA	NA	NA	(2 796)	(22)	NA	NA
							[1–128]	[1–153]		
RF Pos		145 (42.3)		152 (63.1)	122 (57.5)	52 (67.5)	192 (58.0)	138 (62.2)	6 (11.5)	
Neg	NA	198 (57.7)		89 (36.9)	90 (42.5)	25 (32.5)	139 (42.0)	84 (37.8)	46 (88.5)	NA
ACPA Pos				190 (55.0)	133 (60.2)	62 (80.5)	235 (70.6)	179 (80.6)	6 (10.9)	
Neg	NA	NA		155 (45.0)	86 (38.9)	15 (19.5)	98 (29.4)	43 (19.4)	49 (89.1)	NA
Smoking ever		258 (58.6)		153 (43)	127 (58)	43 (55.1)	176 (54.5)	111 (51.4)	27(56.3)	10 (41.7)
Never		182 (41.4)		202 (57)	92 (42)	35 (44.9)	147 (45.5)	105 (48.6)	21(43.8)	14 (58.3)
HLA-DR SE Pos		176 (44)		>200						
Neg	NA	222 (56)		missing data	NA	NA	Not known	Not known	NA	NA
^$^TJC		1^a^	5	9	9	11	0	00	5	7
	NA	(2)	(5)	(11)	(12)	(12)	0	[0–5]	(9)	(14)
		[0–27]	[1–11]	[0–28]	[0–31]	[0–28]	[0–5]		[0–25]	[0–44]
^$^SJC			3	5	5	4	0	0	1	4
	NA	NA	(3)	(7)	(8)	(7)	0	0	(4)	(6)
			[1–13]	[0–22]	[0–22]	[0–19]	[0–5]	[0–2]	[0–13]	[0–11]
^$^CRP		4.3	4	9	11	8	0	0	4	1
(mg/L)	NA	(6)	(26)	(24)	(23)	(27)	0	0	(13)	(7)
		[0–67]	[1–77]	[1–228]	[0–228]	[0–122]	[0–34]	[0–30]	[0–163]	[0–135]
^$^DAS28(4)				4.6	4.7	4.8	1.6	1.5	3.5	3.2
−CRP	NA	NA	NA	(2)	(2)	(2)	(1)	(1)	(2)	(3)
				[1–8]	[1–8]	[2–8]	[1–3]	[1–3]	[1–7]	[2–5)

Data are presented as Numbers (%) or ^$^median (IQR) [min to max]. Note, there are missing data for most variables.

a Tender joint count 78 was use in the arthralgia cohort.

b Duration of symptoms in weeks in early IA but months for pre-RA. Duration of disease in years for remission. Duration of disease in years for OA.

### Flow cytometry results

The gating to report data (% of CD4+ T cells) uses simple classic 2D strategies (Supplementary Fig. S2) as previously reported [14]. We also present a more advanced analysis (UMAP/tSNE maps) for multiple marker expression, a cluster analysis for the presence of subcategories of cells and a SPADE analysis to highlight the ontogeny of subsets in Supplementary Fig. S3.

### Validation of previous model replicating the predictive value of T-cell biomarkers

We used a forward regression strategy to reduce the number of significantly contributing predictors, directly comparing models with/without adding the T-cell biomarkers.

Outcomes predicted:

- progression to IA Supplementary Table S1;
- RA classification Supplementary Table S2;
- MTX-induced remission Supplementary Table S3.

**Figure 1:**
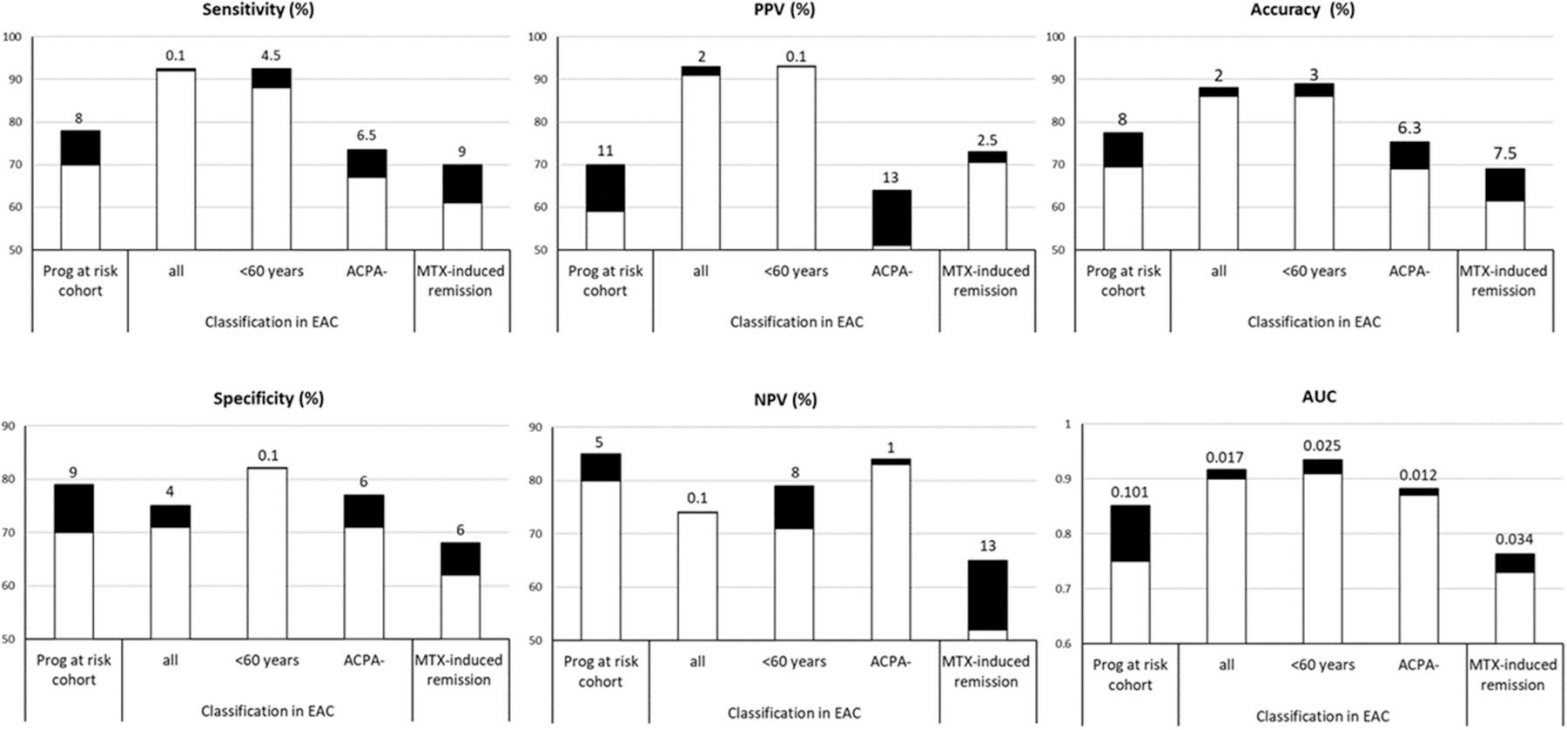
Summary of gain in performance indexes for logistic regression models predicting outcomes across the RA continuum. Sensitivity, specificity, accuracy, positive and negative predictive values (PPV/NPV) were calculated from the model performance matrices and from individual patient probability calculated by the model for AUC. Open bars represent the index values from the regression for clinical data-only models and the black bar above indicates the gain in performance (+ value as %) of these indexes for the model with clinical + T-cell biomarkers. Each stage of the RA continuum is described alongside the *x*-axis

Results are summarized in Fig. 1, presenting the gain in performance indexes when adding T-cell biomarkers (black) over clinical data only (white) to each model. All details of this analysis are presented and discussed in the supplementary materials. AUC are presented in Supplementary Fig. S4. A summary figure of the predictive value of T-cell subsets is also included after the models.

### Progression to inflammatory arthritis in ACPA± at-risk individuals without clinical synovitis

**Figure 2:**
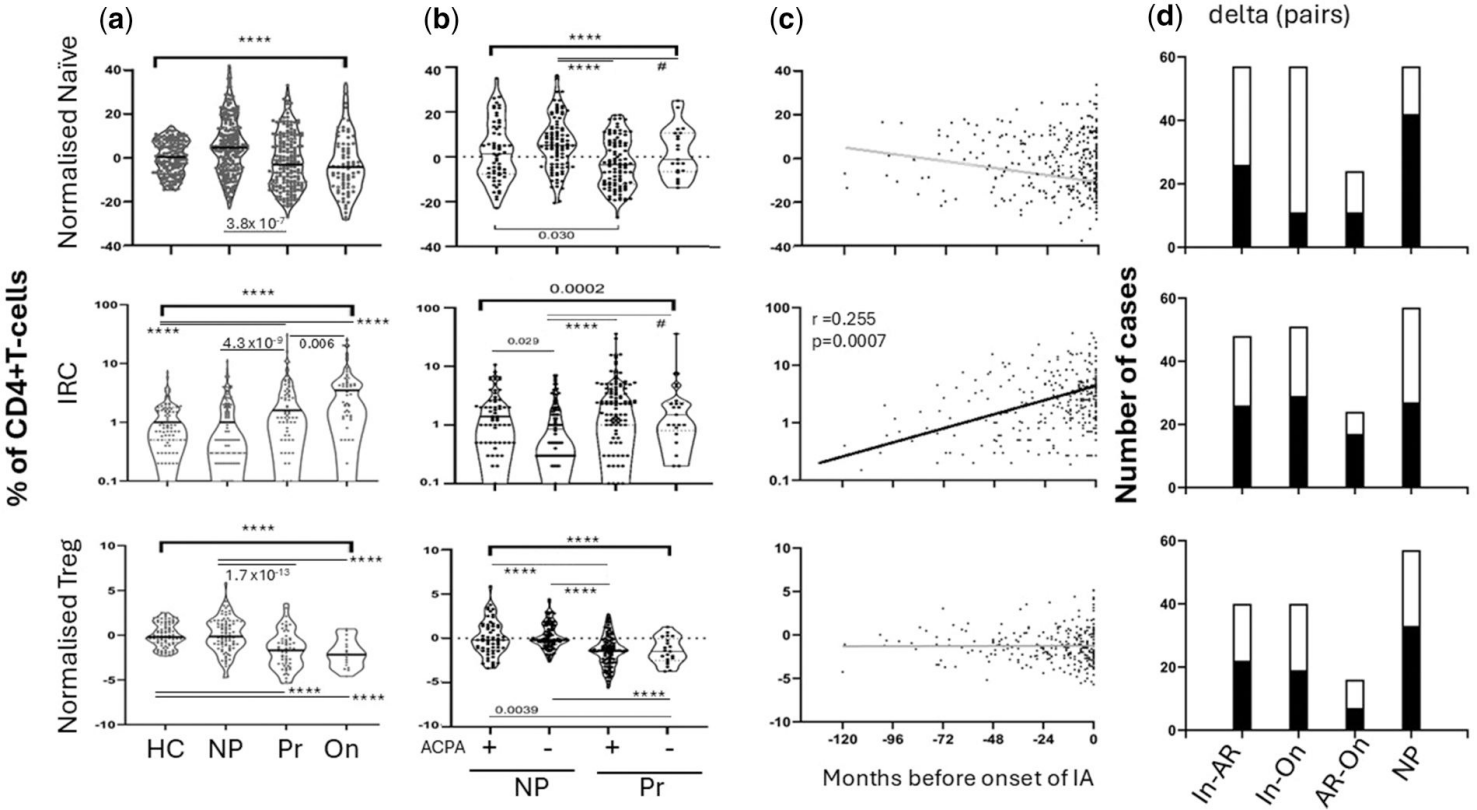
T-cell biomarker data for naive (normalized), IRC and Treg (normalized) in the at-risk of RA, ACPA+ cohort. **a**) T-cell biomarker data displayed in violin plots comparing heathy control (HC, *n* = 174 for naive/IRC and *n* = 120 for Treg), progressor (Pr, *n* = 198) and non-progressor (NP, *n* = 244) ACPA+ at-risk participants and in Pr at time of onset of disease (*n* = 77). **b)** A total of 278 at-risk participants were tested using a research CCP test. T-cell biomarker data are displayed comparing PR to NP, either positive or negative for the CCP test. **c)** Distribution of T-cell biomarkers are displayed in Pr only (*n* = 198) over time, retrospectively calculated with respect to onset of inflammatory arthritis (IA). Correlations were only significant for IRC with a trend for naive cells. **d)** Changes in T-cell biomarker data in paired samples [number of patients showring increase (black bar) and reduction (open) in T-cell biomarker] between inclusion/annual-repeat (In-AR, *n* = 57), annual-repeat/onset of disease (AR-On, *n* = 24), and inclusion/onset (In-On, *n* = 57) as well as in NP (*n* = 53). Thick lines indicate ANOVA and thin lines MWU following Bonferroni correction with *P*-values (****P < 0.0001). Spearman correlations were calculated (best line of fit are displayed), with rho coefficient and *P*-value

All three biomarkers were significantly different compared with healthy controls (HC, *n* = 174), progressors (Pr, *n* = 198), non-progressors (NP, *n* = 244) and data at onset of clinical synovitis (Fig. 2a, ANOVA *P < *0.0001). There was a significant difference between Pr and NP (*P = *3.8 × 10^−7^), but not between Pr and onset of clinical synovitis (except for IRC *P = *0.006) nor between NP and HC.

We did not observe any association between T-cell biomarkers and demographic/clinical data, although trends were seen between age and naive/Treg data (Supplementary Fig. S5).

Results from a citrulinated peptide (CCP) research test for a repertoire of citrullinated peptides particularly associated with the risk of progression were available [24] in 278 patients. A total of 117/278 (42%) were negative and provided a relevant comparison group. Only 21/117 (18%) of patients progressed in the negative group compared with 100/121 (82%) in the positive group (X^2^, *P = *2.3 × 10^−13^). The distribution of T-cell biomarkers between positive/negative Pr and NP was significantly different (Fig. 2b, ANOVA *P < *0.0002). Pr showed no significant difference associated with CCP-research positivity. A difference between NP-ACPA± was observed only for IRC, suggesting that the T-cell subset association is indeed with progression and not with CCP research test results. To validate this conclusion, we tested the added value of the T-cell biomarkers for predicting progression over that of, or in combination with, the CCP-Second Gen test. Logistic regression selected Treg as the best predictor, followed by CCP-Second Gen, then naive T cells, and finally IRC with an overall 75.5% accuracy, AUC = 0.841 (Supplementary Fig. S4). This was better than the CCP-Second Gen alone (65% accuracy, AUC = 0.720) or the 3 T cell biomarkers together (73% accuracy, AUC = 0.807), confirming the additional independent predictive value of combined data (+11% accuracy/+0.164 AUC).

Variations in T-cell biomarkers could occur over time, and we analysed this using annual repeat (AR) visits or at time of onset of clinical synovitis. The distributions of values over time did not change for Treg, while we observed an increase over time for IRC (Fig. 2c, plotting data backward with respect to onset, rho=+0.225, *P = *0.0007) and a reduction trend for naive T cells (rho=−0.155, ns). Paired samples were analysed to assess whether variations in T-cell subsets happen consistently over time (Fig. 2d). Changes were calculated between pairs at inclusion/annual repeat (IN=>AR, *n* = 57), annual repeat/onset (AR=>On, *n* = 24), and inclusion/onset (In=>On, *n* = 57). More reductions were observed for naive T cells [90/138 (65%)], notably over longer periods of time [inclusion-onset 40/57 (70%)]. Despite normalization over time of some high IRC values at inclusion, an increase was seen for IRC in pairs the closest to onset [AR=>On, 17/24 (71%)] compared with during follow-up (45%). For Treg, no trend was observed, confirming the cross-sectional data (∼50% increase/reduction). These results suggest that naive T-cell frequency may continue to reduce over time in Pr, while IRC peak closer to the time of onset of clinical synovitis. In contrast, in 57 In=>AR pairs in NP, an increase over time was seen for naive in 56/77 (73%) cases and for Treg [33/58 (57%)], and a reduction of IRC in 32/57 (57%) cases was seen, with normalization of all high values at inclusion.

### RA classification in an early arthritis clinic (EAC)

T-cell biomarkers were also different between EAC outcomes (Fig. 2a, ANOVA, *P < *0.006). A reduction in naive T-cell frequencies in the RA and UA=>RA groups was observed. Higher frequencies of IRC were associated with PsA, but all disease groups had higher IRC than HC, though insignificantly. Treg were reduced in UA=>RA/RA. Comparing RA (Fig. 2b, *n* = 302 + 77) to non-RA (*n* = 126), only naive (MWU, *P = *0.0009) were significantly reduced in RA while IRC/Treg were borderline.

**Figure 3:**
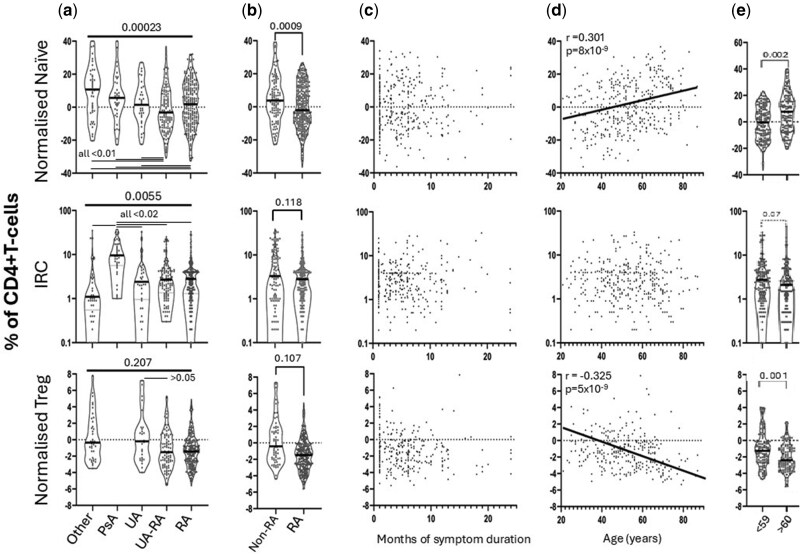
T cell biomarker data for naive (normalized), IRC and Treg (normalized) in an early arthritis clinic cohort. **a)** T-cell biomarker data are displayed comparing various outcomes after 24 months follow-up: 238 patients had RA at baseline and 64 evolved to RA over time, 37 remained undifferentiated arthritis, 44 developed psoriatic arthritis, 45 had others diagnoses (non-persistent inflammation, reactive arthritis, gout). **b)** T-cell biomarker data are displayed comparing RA & UA-RA (*n* = 302) versus all others as non-RA (*n* = 126). **c)** Distribution of T-cell biomarkers is displayed over duration of symptoms at the time of assessment in RA patients (*n* = 283 due to missing data). No correlations were observed. **d)** Distribution of T-cell biomarkers is displayed with respect to age (years) in RA patients only (*n* = 302). Correlations were only significant for naive cells and Treg. **e)** T-cell biomarker data are displayed comparing RA patients below (<59, *n* = 189) and above (>60, *n* = 113) the age of 60 years old. Thick lines indicate ANOVA and thin lines MWU following Bonferroni correction with *P*-values. Spearman correlations were calculated (best line of fit are displayed), with rho coefficient and *P*-value

We investigated data in the RA group (*n* = 302 from EAC and 77 at onset of clinical synovitis) for association between T-cell biomarkers and other parameters (Supplementary Fig. S5). There was no association with gender, smoking status or with classification parameters (RF, CRP, joint counts), although lower naive T cells were observed in ACPA+ RA (unadjusted MWU *P = *0.012). Symptom duration did not affect T-cell biomarkers (Fig. 3c), suggesting that disturbances in T-cell subsets are likely to be established during the pre-clinical phase of RA. In contrast, trends for correlations between age and the naive or Treg biomarkers were observed (Fig. 3d, Rho=+0.301, *P = *8.1 × 10^−9^ for naive T cell and rho=−0.325, *P = *5.2 × 10^−9^ for Treg).

Both correlations appeared driven by a group of patients aged >60 years, not mirrored by a decline in younger patients. We separated RA developing in older patients as late-onset RA (Fig. 3e, >60 years, *n* = 113, median age 68 years, range 60–82) *vs* younger RA patients (*n* = 266, median age 47, range 21–59). Naive T cells were lower in the younger RA group (median 0.2%) than in the late-onset RA group (+7.2%, *P < *0.0001). This was also observed for Treg (*P = *0.0001) where late-onset RA patients had lower Treg (MWU *P = *0.001). IRC were higher in the younger RA group (*P = *0.071). The age relationships with time disappeared, suggesting they indeed represent different groups. RF positivity in late-onset RA patients (43%) was less frequent than in younger RA patients (68%, X^2^  *P = *0.00011) as previously reported [25]. In contrast, ACPA positivity was 80% in the younger patients and 74% in late-onset RA. Other parameters (CRP/SJC-TJC/duration/smoking status) were not different. This is suggesting possible over-classification based on the presence of ACPA autoantibodies in >60-year-old patients.

Of note, regression models for RA classification in >60 and <60-year-old participants are described in Supplementary Table S2. Only ACPA and SJC were predictive of late-onset RA (with no value for T-cell biomarkers), while the model in younger patients performed better than in the overall group, while selecting the same variables (Fig. 1).

### Response to MTX±TNFi

**Figure 4:**
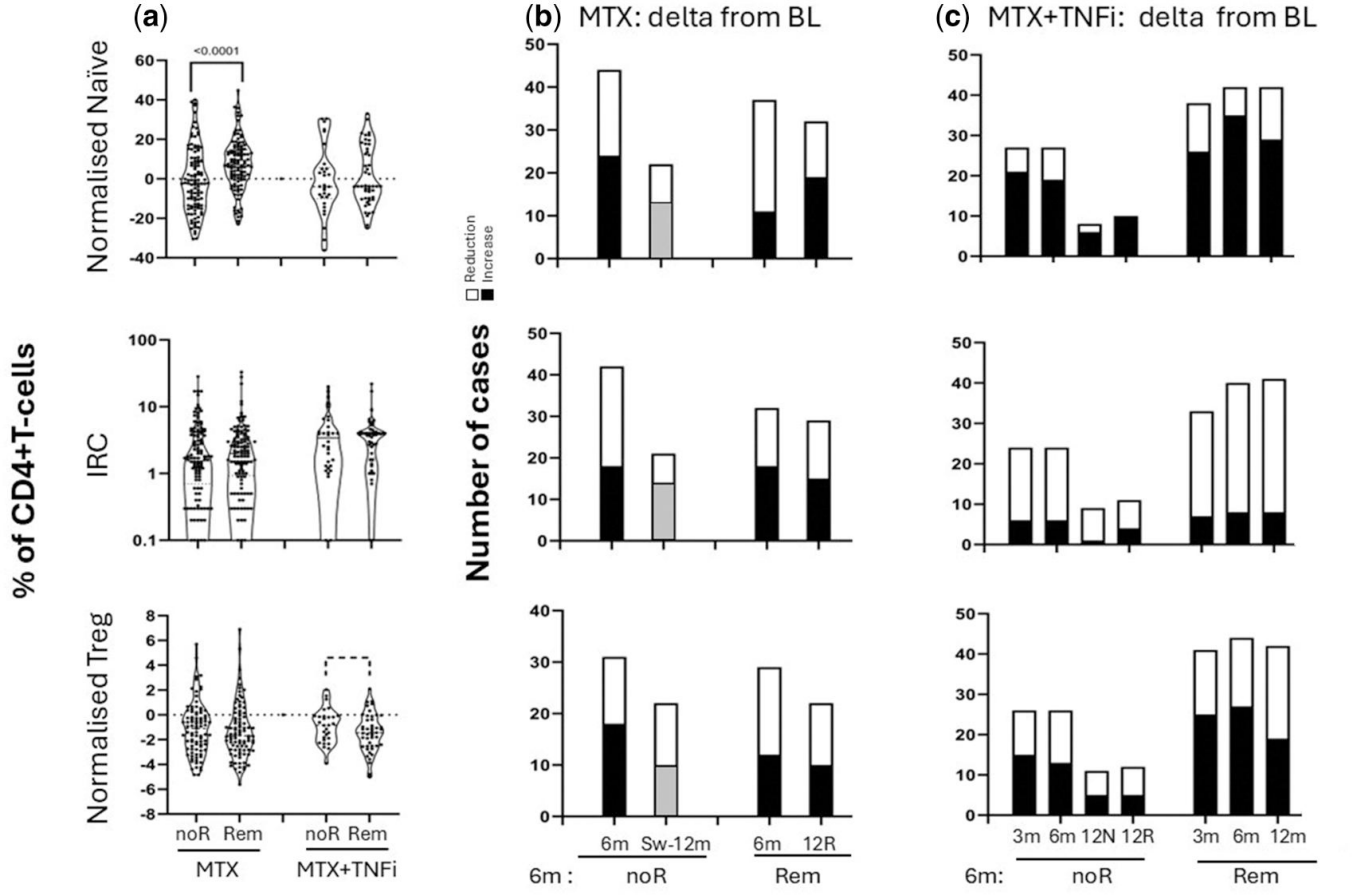
T-cell biomarker data for naïve (normalized), IRC and Treg (normalized) in early RA patients treated with methotrexate (MTX) or a TNF-inhibitor (TNFi). **a)** T-cell biomarker data are displayed comparing two outcomes [no remission (noR) and remission (rem) after 24 weeks] in early RA patient treated with MTX (*n* = 221) or MTX+TNFi (*n* = 78). **b)** Number of patients showing increase (open bar) and reduction (black) in T-cell biomarker data in early RA patients treated with MTX compared 6 months to inclusion, stratifying patients by outcome at 6 months (6m) and achieving remission (Rem, *n* = 42) versus no-remission (noR, *n* = 52) defined by DAS28 <2.6. After 6 months, some patients not achieving remission were switched to TNFi and increase/reduction were then calculated at 12 months compared to baseline (Sw-12m). In patients achieving remission at 6 months, measures were repeated at 12 months and patients separated based on remaining in remission (12R) or not (12N). **c)** Number of patients showing increase (open bar) and reduction (black) in T-cell biomarker data in early RA patients treated with MTX+TNFi comparing data at inclusion and 3, 6, 9 or 12 months later, stratifying patients by outcome at 6 months (6m) as achieving remission (Rem, *n* = 48) versus no-remission (noR, *n* = 30) defined by DAS28 <2.6. Of note, most but not all patients had data/samples at all the follow-up time points.

We combined patients from the two clinical trial arms (*n* = 78) of whom 48/78 (61%) achieved remission at 6 months. There was no difference in naive, Treg and IRC frequencies between outcomes (Fig. 4a), despite a lower Treg median in remission. No clinical variable was associated with achieving remission (Supplementary Table S4) and no model could be computed, confirming the still unmet need for biomarkers to predict response to TNFi.

### MTX effect on T-cell subsets

The effect of MTX treatment on T-cell biomarkers was analysed over time (*n* = 94), separating patients based on achieving DAS remission at 6 months. For patients in remission, treatment remained stable until 12 months (*n* = 42). In the non-remission group (*n* = 52), patients were escalated to various combinations of csDMARDS after 6 months, some with samples at 12 months (*n* = 27). We analysed paired samples by calculating changes (delta) in T-cell biomarkers at 6 or 12 months (Fig. 4b). No clear direction of change for naive T cells was seen in the non-remission group at 6 months (24 increases/20 decreases). For patients achieving remission, naive T cells showed more reduction initially (11 increases/26 decreases, *P = *0.025 compared with patients not achieving remission); however, the tendency was inverted at 12 months (19 increases/13 decreases, *P *= 0.014 compared with 6 m) suggesting MTX may need time to affect naive T cells. IRC continued to increase in both groups at 6 months (*P = *0.250) and did not change direction after 12 months in the remission group. No consistent direction of change was observed for Treg (*P > *0.200) at 6 or 12 months.

### MTX±TNFi effect on T-cell subsets

A similar analysis was performed of patients receiving MTX+TNFi. In the non-responder group [*n* = 27/70 (38%)], eight patients achieved remission late at 12 months and many withdrew from the trial (no samples). In the remission group at 6 months [44/70 (62%)], four flared before 12 months. Analysis of the change over time (3 m, 6 m) showed consistent increases in naive T cells in both remission [Fig. 4c, 35/42 (83%)] and non-remission [19/27 (70%), *P = *0.570] at 6 m. IRC clearly showed more reductions in both groups (>70%). We observed more increases in Treg in remission [27/44 (61%) at 6 months] than in non-remission [13/26 (50%)], but not significant (*P = *0.355), with no noticeable change at 12 months.

Importantly, 22 patients who failed MTX at 6 months received TNFi and all went into remission at 12 months. An increase in naive T cells was observed in 13/22 (60%) (Fig. 4b, Sw-12m, grey histogram). In contrast, IRC were no longer being reduced by TNFi and continued to rise (67% increase). Treg showed no longer a clear increase either (55% increases).

### Established disease patients achieving remission (on drug)

T-cell biomarkers were first compared between remission on either cs- or bDMARDs.

**Figure 5:**
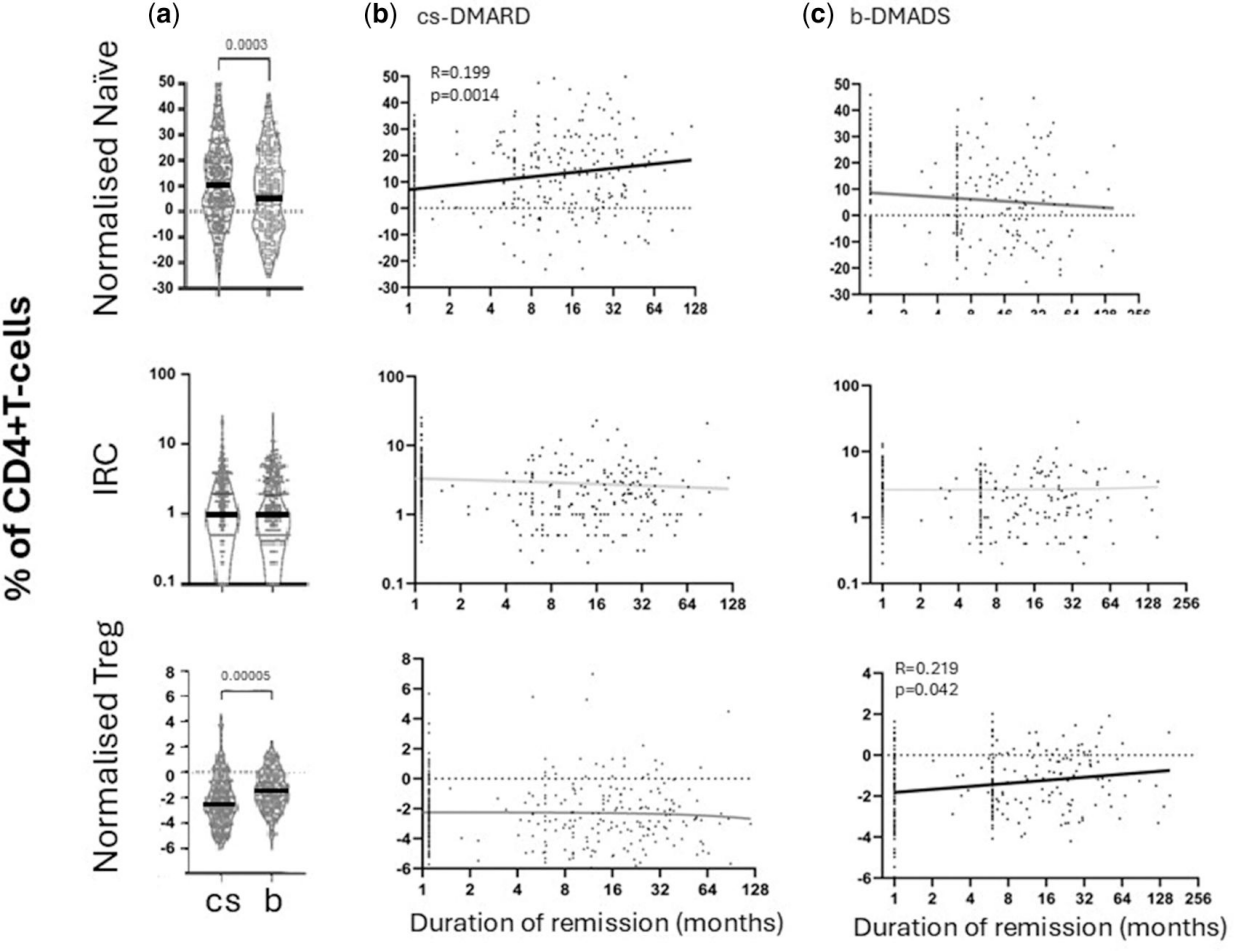
T-cell biomarker data for naive (normalized), IRC and Treg (normalized) in a cohort of RA patients (*n* = 683) achieving clinical remission based on DAS28 < 2.6. **a)** T-cell biomarker data are displayed comparing patients achieving remission defined by DASD <2.6 on classic-synthetic (cs) DMARDs (*n* = 418) or on biologic (b) DMARDs (*n* = 265). MWU *P*-values are displayed on the graph. **b)** Distribution of T-cell biomarker data are displayed in RA patients over time, prospectively calculated from first time in remission and treated only with cs-DMARDs (*n* = 418). **c)** Distribution of T-cell biomarker data is displayed in RA patients over time, prospectively calculated from first time in remission and treated only with b-DMARDs (*n* = 265). Lines indicate MWU test with *P*-values. Spearman correlations were calculated (best line of fit are displayed), with rho coefficient and *P*-value

Higher naive cells were observed in csDMARDs remission (Fig. 4a, *n* = 418, median +10%) compared with remission on biologic (*n* = 265, +1%, *P = *0.0003). This was associated with a possible trend for increase in naive cell over the duration of remission (Fig. 5b, rho = 0.200, *P = *0.0014) from first achieving DAS28 < 2.6 to stable remission for several years. In contrast, no effect on naive T cells was seen over time in bDMARD remission (Fig. 5c). There was no difference in IRC frequencies between drugs; however, there was a reduction of IRC when comparing first time in remission to stable/long-term remission on csDMARDs (data not shown, *P = *0.0031), not seen directly as a correlation and not seen in remission on bDMARDs. Finally, Treg were higher in bDMARD remission (*P = *0.00005), in a relationship over time (rho=+0.219, *P = *0.042) and this was not seen in csDMARDs.

## Discussion

Our main objective was to understand the dynamics and relationships of circulating T-cell biomarkers compared with those currently in use in clinical practice in a large number of participants across the RA continuum. We described the dynamics of the three T-cell biomarkers over time and established their relationship with demographic/clinical parameters in ACPA+ at-risk Pr and over RA classification (with respect to research CCP-Second Gen ACPA tests and with ACPA-negative early IA). We demonstrated their independence from other biomarkers currently used to predict RA outcomes and validated their added value (discussed in detail in the supplementary materials). We also established that naive and Treg cells change in patients above the age of 60, possibly due to concomitant osteoarthritis (OA), allowing us to refine the population in which these biomarkers should be used.

### At-risk phase

All three biomarkers were highly disturbed and had important predictive value for progression. Only age appeared to influence naive/Treg while not interfering with the prediction of progression. While not significantly decreasing over time as a group, naive cells in paired samples decreased in Pr (56% of pairs) while they increased in NP (83%). This suggested that dynamic changes continue over the at-risk phase in the RA continuum for naive T cells. Switching from low- to high-risk categories over time (based on 80% specificity cut-off as previously defined [14]) therefore justifies repeat analysis (annually at least). Treg decreased in Pr, but only in 42% of pairs (no trend in NP) with no relationship with time. IRC were previously associated with progression >12 months [21]. Here, a cross-sectional analysis confirmed that IRC increase closer to the onset of IA and overall, in 65% of paired samples, whilst not in NP. Monitoring IRC over time in patients with a high risk could provide further stratification toward intervention studies, where patient selection based on an imminent rather than overall risk could reduce the need for follow-up time.

### Classification of RA in patients with early arthritis

We confirmed that T-cell biomarkers are independent predictors of fulfilling RA classification, particularly useful in ACPA patients, and are not correlated with other clinical parameters. However, age affects how these biomarkers can be used. While the term ‘late-onset RA’ (LO-RA) refers to patients who developed RA after the age of 60, other differences with RA (occurring in mid-life) exist. The female-to-male ratio tends to be less pronounced in LO-RA (in our cohort, 61% compared with 75% in the younger group). There is a lower proportion of RF+ in LO-RA individuals (43% in LO-RA *vs* 68%). Joint count records do not detail large/small joints while LO-RA usually involves more large joints. T-cell biomarkers may provide new insights into difference in pathophysiology between diseases, as naive T cells are clearly higher than expected in LO-RA, Treg are reduced and IRC are not particularly raised. The normalization procedure that we applied allows the effects of age to be accounted for (increase/reduction above expected normalized values), allowing for other effects to be addressed directly. Raised frequencies of naive CD4+ T cells and loss of Treg were previously observed in OA patients [26]. In older individuals, positivity for RF also increases with age [27, 28], while ACPA positivity is also observed in ∼6% of OA patients [29, 30]. This suggests that OA rather than age-related immunological changes is a confounding factor for RA classification in ACPA+ patients >60 years old. In either case, the mechanism being the increase in naive T cells in OA remains to be explained when thymic activity clearly decreases with age [6, 7].

### Predicting response

Previous results for the prediction of MTX-induced remission (naive CD4+ T cells only) were replicated and the lack of value for TNFi response was clearly established. This provides a rationale to stratify early, drug-naive RA patients unlikely to respond to MTX for initial treatment with TNFi. This will require randomized control trials to establish a clear gain in preventing long-term effects of RA when treated more aggressively with TNFi.

### Drug effect on the biomarkers

In early RA patients achieving MTX-induced remission, naive T cells initially continued to decrease, in contrast to the non-remission group, where changes did not have a particular direction. With time, MTX tended to reverse this tendency and naive cells increased. MTX treatment is not able to effectively control IRC, regardless of disease activity, and Treg appears to be a relatively stable biomarker.

In contrast, over the course of TNFi treatment, naive T cells increased irrespective of remission, suggesting a direct drug effect. The effect of TNF inhibition on the activity of the thymus was not directly investigated here, but there are reports that TNF-alpha is constitutively expressed in the thymus [31, 32]. The thymus, however, should also produce new Treg, but this effect was not seen. The pool of circulating Treg consists of both thymic Treg (t-Treg) and inducible Treg (i-Treg) generated in the periphery. Gut dysbiosis present in pre-clinical RA [33–37] may in part be responsible for altering the profile of immuno-regulatory metabolites notably responsible for the generation of i-Treg [38–41]. TNFi is believed to restore a ‘healthier’ gut microbiota [42], and it would be relevant to include a marker such as Helios (only expressed in t-Treg) to examine this issue further.

Importantly, the loss of TNFi-mediated effect on IRC, when used after failing to respond to MTX-alone, suggests that a major opportunity to control inflammation-related effects on T cells is missed when the use of TNFi is delayed. This should also be considered when rationalizing the use of T-cell biomarkers to decide between MTX alone or MTX+TNFi.

### RA remission

Patients in remission on csDMARDs had higher naive cells compared with those on bDMARDs, possibly due to MTX only being able to produce remission in the less dysregulated individuals. Naive cells, however, increased over time in csDMARD remission, suggesting that long-term control of disease activity may allow recovery of naive cells. Thymic activity is likely responsible for this slow effect, especially in light of previously reported data on naive T-cell recovery in csDMARD remission [43, 44] and the stability of the remission stages [14]. The effect of TNFi on increasing naive T cells during the establishment of remission indicates further stabilization over time in remission.

IRC in remission are clearly lower compared with active disease; however, this also occurs slowly on csDMARDs (higher at time of first achievement of remission), while rapidly in bDMARD-treated patients. Once in stable remission, patients often desire to reduce their treatment although the risk of relapse is significant and requires careful management [45]. Relapse in remission was associated with higher IRC [14, 18, 43] and the strongest predictor of flare in patients tapering csDMARDs [18]. This suggests that tapering should not be offered until IRC are clearly decreased.

IRC are quickly reduced by bDMARDs; however, they showed no predictive value for flare when patients remain on treatment [20], while they were predictive of flare when tapering [20]. The value of IRC when offering tapering is therefore independent of the type of treatment.

Treg were higher on bDMARDs, and this was seen from initiation of TNFi-induced remission and continued over the course of achieving stable remission. This is specific to bDMARDs and was previously associated with ability to sustain remission when tapering bDMARDs (best predictor) [20]. Monitoring Treg therefore also has important biomarker value for recommending tapering of bDMARDs.

### Biological mechanisms

Patients ACPA+ at-risk who later developed RA showed CD4+ T subset disturbances (increasing the closest to disease onset for IRC), while there appear to be limited changes later, once disease has initiated, suggesting that such dysregulations are acquired during the pre-clinical stage and rather a stable event while associated with response to MTX. Whether disturbances of these subsets are merely biomarkers or whether they participate to the progression towards RA remains an open question. We reported dysfunctional features in naive CD4+T cells in early, untreated RA, notably in relation to a decline in thymic activity, the differentiation of IRC (under a putative IL-6 drive), while response to antigenic challenges suggested maintenance of the naiveite in both IRC and naive cells [6], while the naive TCR repertoire is shaped by HLA alleles [46]. Progression to RA in at-risk individuals has been associated with systemic inflammation (IL-6/TNF, chemokines) and transcriptional change in naive T cells, suggesting activation while remaining naive, and potentially autoreactive naive CD4+ [47]. These data reinforced the concept of a non-immunological/non-antigenic challenge affecting naive CD4+ T cells. Inflammation is a well-known trigger of epigenetic changes, suggesting a mechanistic mean to enable the changes observed in naive T cells. We showed that epigenetic remodeling specific to naive CD4+ T cells occurs in early RA, centered on a network of modifications driven by IL-6/TNF signalling, in a subpopulation of naive cells resembling IRCs [48]. Recently, the accumulation of epigenetic changes in naive CD4+ T cells in at-risk individuals over the course of progression to clinical onset was described [49]. Notably, CD62L was involved which is the main marker differentiating naive CD4+ T cells from IRC [48] and their increased resistance to apoptosis [43]. Our data, combined with the effectiveness of Abatacept in preventing RA development [50–51], strongly support the concept that T cells are the central orchestrator of the events leading to RA, while how pre-clinical inflammation develops remain to be elucidated. Microbiota dysbiosis observed preclinically in the mouth [52] or in the gut [53–54] (possibly increased gut permeability/systemic inflammation) offers possible clues to explore, while this may also be related to the loss of Treg via lowering nutrition-generated inducible Treg and/or increasing Th17 differentiation [55] as also seen epigenetically in early RA [48] and in at-risk individuals [49]. More work is needed to identify the origin of the defect in Treg, differentiating between loss of naturally occurring Treg, or inducible Treg or tissue-resident Treg.

### Limitation

Although the models we built suggest the added value of including T-cell subsets at each step of the RA continuum, there are limitations in using regressions. This is usually associated with handling of missing data, using multiple imputation, a recommended approach that reduces imprecision and bias compared with complete-case analysis or single-value imputation methods (even using multiple imputation) [56]. By drawing on the statistical properties of the dataset (distributions and inter-variable relationships), multiple imputations produce more robust estimates and reduce the likelihood of biased or misleading conclusions. Furthermore, we were careful to not use clinical variables with a significant amount of missing data (GH, HLA-DR-SE). For T-cell biomarkers, missing data affected Treg more than naive/IRC while the numbers of cases for each outcome were sufficient to allow for adequate imputations.

These biomarkers remain research tools at this point in time. Prospective studies are needed to demonstrate outcomes of (i) using TNFi in patients predicted to fail response to MTX; (ii) preventing imminent progression by stratifying at-risk individuals for intervention with anti-T-cell co-stimulation, for example; and (iii) offering tapering in patients on bDMARDs achieving a low-risk state. Such findings would support the value of these biomarkers for improved disease management and provide a rationale for economy in drug usage/tapering. Full external validation in an independent cohort remains the next critical step, while replication efforts in biomarker research are often challenged by heterogeneity in cohort selection criteria and/or outcome definitions worldwide.

Furthermore, to adopt this more widely, we developed a dry-tube (DT) approach to staining cells, which would facilitate transferring the technology to standard flow cytometry services. We performed a validation of these antibody pre-coated/bar-coded tubes in supplementary material. We demonstrated the feasibility of the DT technology for two key CD4^+^ T-cell subsets (naive and IRC), benchmarked against conventional wet-tube (WT), showing equivalent distribution and no bias for naive T-cell enumeration (see supplementary data). IRC values showed bias at high frequencies, primarily in the clinically relevant range reflecting actual improved subset separation in the DT panel (e.g. superior resolution), which is advantageous for routine NHS laboratory workflows. The lack of signal in DT technology for Treg panel suggest incompatibility of the drying chemistry with intracellular FoxP3 staining. Acceptable detection of ‘putative’ Treg by surface markers only was nonetheless verified. These features collectively support DT suitability for daily clinical practice and underscore its applicability for routine NHS laboratory use.

### Conclusion

This study highlights the potential of CD4+ T-cell subsets as biomarkers for predicting RA progression, developing prevention strategies, classifying early RA, predicting MTX treatment response and rationalizing the use of TNF inhibition early to avoid damage, and counselling patients on tapering bDMARDs. The findings also suggest that coexisting conditions like OA can influence T-cell subset profiles, and confirmed that different DMARDs have distinct effects on these immune cell populations, shaping the immunological state of patients with RA.

## Supplementary Material

keag286_Supplementary_Data

## Data Availability

Fully anonymized data are available via the corresponding authors based on reasonable request and subject to agreement with the University of Leeds.
