# Supplementary material for: T-cell subset biomarkers across the rheumatoid arthritis disease continuum: from clinical utility to adoption in daily practice
Source: Rheumatology (Oxford). 2026 Jun 10;65(6):keag286. doi: 10.1093/rheumatology/keag286 (PMC13303292; doi:10.1093/rheumatology/keag286)
Supplement: keag286_Supplementary_Data [file keag286_supplementary_data.docx]

**Supplementary material**

***Details Patient group description***

Ethical approval was obtained from National Research Ethics Committees at different phases of the IA-continuum (National Research Ethics Service, West Yorkshire Ethics Committee: REC09/H1307/98, REC10/H1307/138, NCT02433184, REC06/Q1205/169). All participants provided informed consent prior to recruitment. Participants included in this analysis were selected based on having T-cells subset data available. Healthy controls (n=174) were used to establish age-associations for naïve and Treg measurements as previously described ^[1, 2]^.

Individuals with arthralgia and no clinical synovitis as established by a rheumatologist and ACPA+, based on a hospital screening CCP-2^nd^ generation test (Bio-Rad Laboratories), were included as the “at-risk cohort” (n=442), as previously described ^[3, 4]^. The clinical endpoint is the development of synovitis on clinical examination at which point patients are withdrawn. Patients were tested with a 3^rd^ generation research test (Quanta Lite CCP3; Inova Diagnostics), as previous described ^[5]^ and classified as CCP-3^rd^ Gen positive or negative, while all were positive by screening CCP-2^nd^ Gen.

Early disease: Patients were selected from our EAC register (2015-2020, n=486). The 2010 EULAR diagnostic criteria were used to classify RA and UA patients who developed RA over 24 months. Alternative diagnoses included UA, PsA, a group with non-persistent symptoms, Ankylosing Spondylitis (AS), Connective Tissue Disease (CTD), gout and reactive arthritis. Patients at inclusion in the register were not on any treatment, including no oral steroid, while intramuscular localised steroid injections were allowed, with no effect on T-cell subsets previously described ^[1]^.

1st treatment: Newly classified RA patients were selected when prescribed MTX (n=221) in a standard of care protocol, starting at 15mg/week and escalating to 25mg/week over 8 weeks, to replicate the original prediction model (n=70) for MTX-induced remission. Additional cs-DMARDs (sulfasalazine or hydroxychloroquine) were allowed if low disease activity was not achieved by 3 months (DAS28<3.2).

A group of early drug naïve RA patients received MTX+Etanercept (n=78), as part of 2 clinical trials (VEDERA and EMPIRE ^[6, 7]^ with 12 months follow-up. 93 patients received MTX alone as standard of care/controls arm of the trials. In both groups, clinical visits were scheduled 3 monthly for 6 month/1 year, although not all visits were associated with a blood sample.

DAS28<2.6 was used to define the achievement of clinical remission in both groups.

RA patients who achieved DAS28-remission (DAS28<2.6) having been treated only with cs-DMARDs only (MTX, sulfasalazine, hydroxychloroquine, n=448) or with combination with bDMARDs (anti-TNF agents, n=265) were included. Any duration of remission was allowed from participant when remission was achieved for the 1^st^ time to several years in stable remission.

Groups Summary : illustrated in SUPP-Figure 1

- **442 ACPA+ at-risk participants** (with musculoskeletal symptoms but no clinical synovitis)
  - 305 had results for an ACPA-3^rd^GEN test
  - 53 with a sample at annual-repeat in non-progressor (NP)
  - 57 with a sample at annual-repeat before progression (Pr)
  - 77 with a sample at onset of IA
- **428 participants from the early arthritis clinic (EAC)**
  - 238 RA at inclusion and 64 developed RA over 24 months (302 total)
  - 37 remained UA after 24 months
  - 44 had early, DMARDs naïve PsA
  - 45 had other diagnoses
    - Altogether 126 non-RA
- **220 early RA patients treated MTX** from the EAC or the control arms of 2 clinical trials
  - 94 with longitudinal samples (6 and/or 12 months)
- **78 RA from the MTX+TNFi arm of the 2 clinical trials**
  - longitudinal samples (0-3-6-9-12 months, not complete time line for all patients)
- **683 RA patients in remission clinic** (achieving DAS<2.6) including patients
  - 418 on csDMADs
  - 265 on bDMARD
- **174 healthy controls**

**Supplementary Figure S1**: Schematic representation of the groups of patients used.


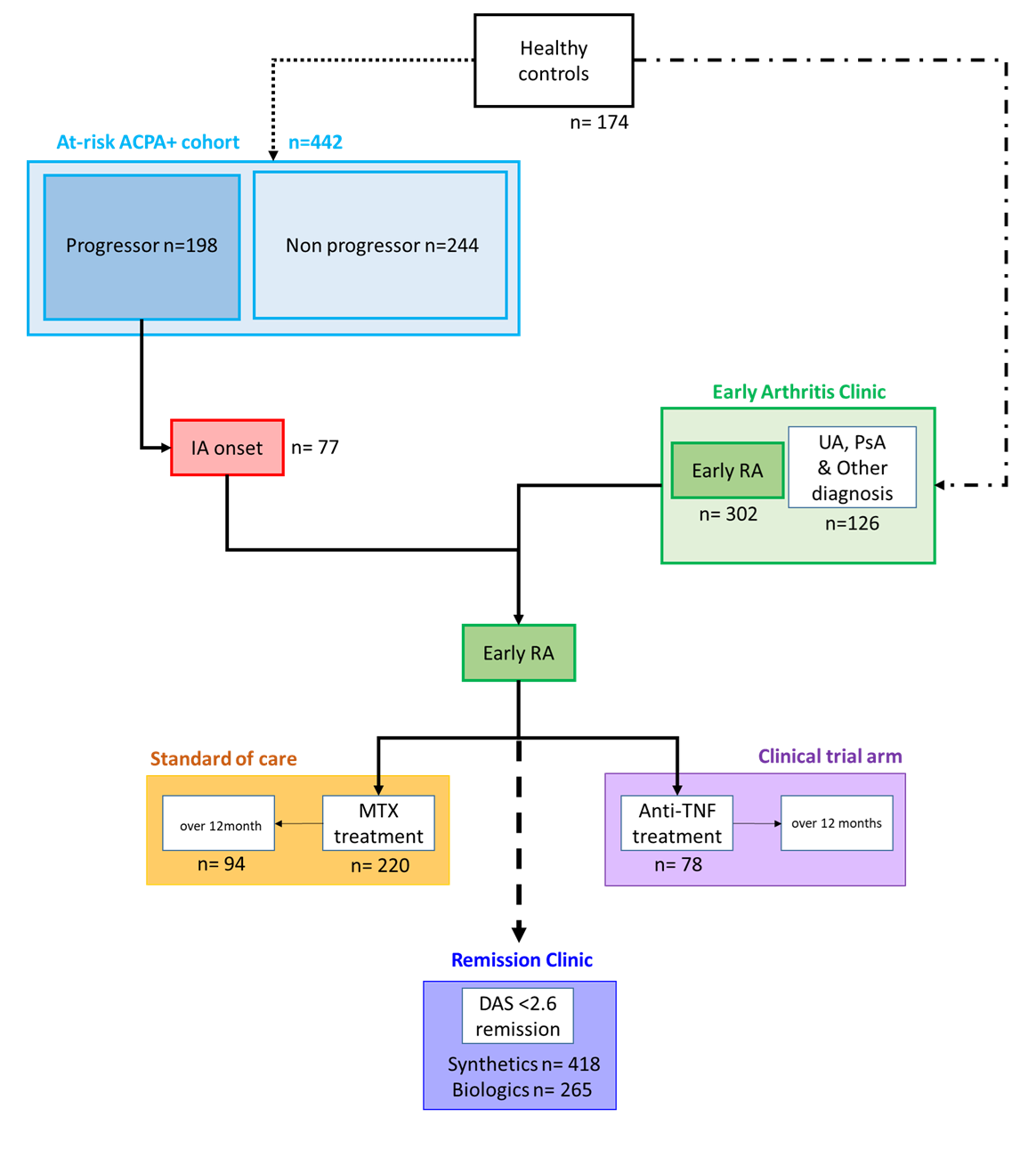


**Flow cytometry methodology**

Peripheral blood was collected into **EDTA** (4 ml) was collected and stored at **room temperature** until processed.

Subset quantification was performed using flow cytometry by the NHS-routine immunology services (in accordance with Good Laboratory Practice). **SOP available on request**.

Briefly, naïve and IRC CD4+T-cell subsets were identified based on their expression of CD45RB-FITC, CD45RA-PE and CD62L-APC. CD4 Treg were quantified by cell surface staining for CD25-APC and CD127-PE followed by intracellular staining for FOXP3-FITC using the anti-human Foxp3 staining kit (Insight Biotechnology, Wembley, UK).

Flow cytometry analysis was performed on a QUANTO cytometer (BD), using BD Biosciences FACSDIVA software. Gating was performed as showed on SUPP-Figure S2A. Subset frequencies were reported as % of CD4+T-cells.

**Antibody clones used :**

| Panel 1: naïve and IRC | Clone | company |
| --- | --- | --- |
| CD4-BV421 | RPA-T4 | BD |
| CD3-V500 | UCHT1 | BD |
| CD45RB-FITC | MEM-55 | Serotec |
| CD45RA-PE | F8-11-13 | Serotec |
| CD62L | 130-091-755 | Miltenyi |
| Panel 2: Treg | | |
| CD4-BV421 | RPA-T4 | BD |
| CD3-V500 | UCHT1 | BD |
| CD25-Pe-Cy7 | 2A3 | BD |
| FOXP3-AF488 | 236/E7 | BD |
| CD127- PERCP-Cy5.5 | M21 | BD |

There is an age relationship between naïve and Treg frequencies as shown in SUPP-Figure S3. IRC were not related to age. We established regression equations using healthy controls data to normalised frequencies of naïve (n=174) and Treg (n=xxx) CD4+T-cells:

**[expected naïve] = -0.63 x [age] +66.6**

**[expected Treg] = +0.061 x [age] +1.83**

Subset frequencies For naïve and Treg are therefore reported as normalised % of CD4+T-cells using the heathy control range:

**[normalised frequency] = [frequency observed in patient] - [frequency expected at that age].**

The latter being calculated from the age-subset frequency correlation described above.

Data analysis using CD4+T- cell numbers for each subset rather than % of total CD4+T-cells were previously discussed and did not impact conclusion or improvement statistics. Therefore we chose not to convert % into cell number to avoid additional manipulation of the raw data.

***tSNE/Umap and SPADE analysis***

tSNE (visualization of t-Distributed Stochastic Neighbour Embedding; Flow-Jo) maps were generated to visualize expression levels of markers on gated CD4+T-cells (SUPP-figure-2B and C). Analysis was performed using the default t-SNE parameters. Clustering analysis of the tSNE output was then performed using both the cluster function of Flow-Jo and SPADE (Spanning-tree Progression Analysis of Density-normalized Events; https://www.cytobank.org/). Clusters of nodes were then labelled manually according to the cell’s phenotype.

**Supplementary Figure S2:**

**A) Classic 2D flow cytometry gating strategy.** Representative flow cytometry plot for naïve (green box, CD45RBhigh/CD45RA+/CD62L+), IRC (red box, CD45RA+/CD62L-) and Treg (blue circle FoxP3+/CD25+/CD127-) following gating on CD3+CD4+ T-cells (purple box). Analysis uses this classic strategy to records data (% of CD4+T-cells).

**B) Multi-marker analysis of cytometry data** : Top plots: U-MAP and tSNE analysis of concatenated data from 10 representative participants based on gated CD4+T-cells and analysis of expression levels of the 3 markers. Of note, the participants chosen for the IRC or Treg cell analysis were selected due to having high % of IRC/Treg to clearly define small sub-populations. Middle plots: individual representation of data for 3 participants highlighting naïve, IRC and Treg subpopulations on U-MAP (naïve) or tSNE plot (IRC/Treg). Bottom plots: Cluster analysis of the concatenated data segregating clusters of cells based on the expression of the 3 markers. Small clusters (blue arrows) are indicated by arrows on 2D plots.

**C) SPADE trees of cellular hierarchy** of CD4+T-cells from the representative patients used in panel-A, showing tree branches that could be clearly identified as naïve (1) , IRC (2) , effector (5 and 9) and memory (7-8-9) cell subsets. Branch 3 and 4 suggest a different subpopulation as their expression of CD45RB was intermediate rather than high like in IRC.

**A**


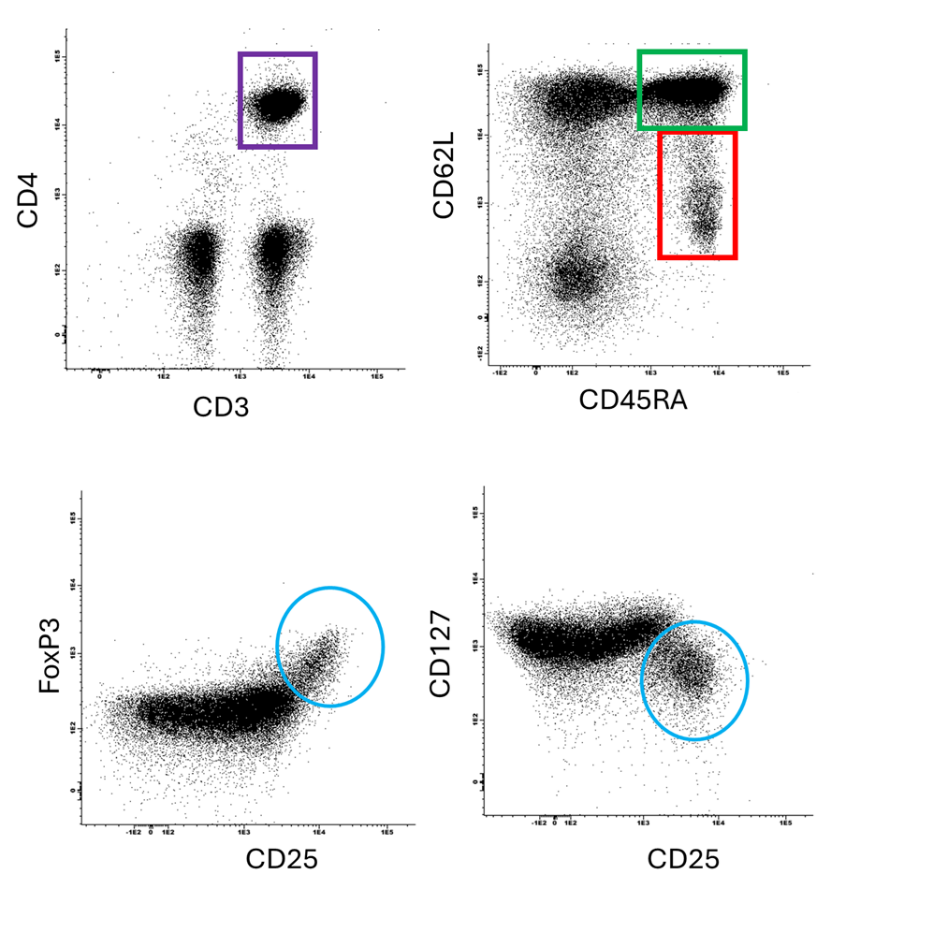


**B**


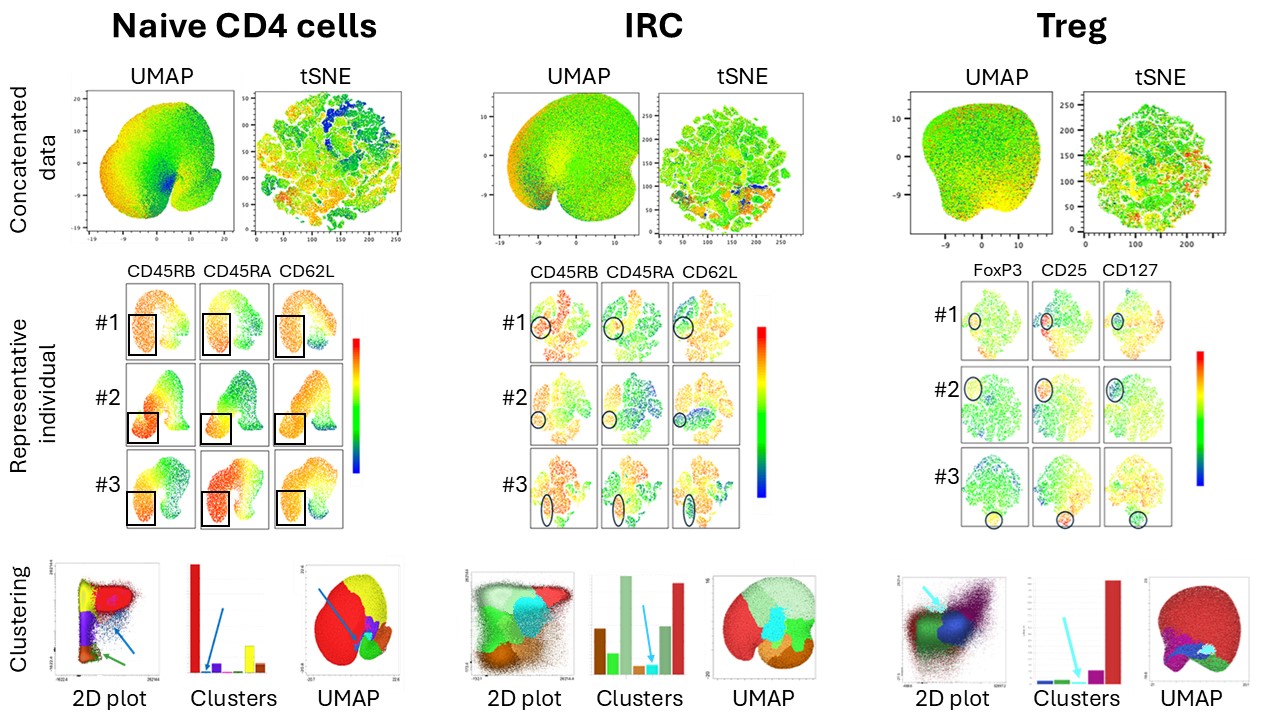


**C**


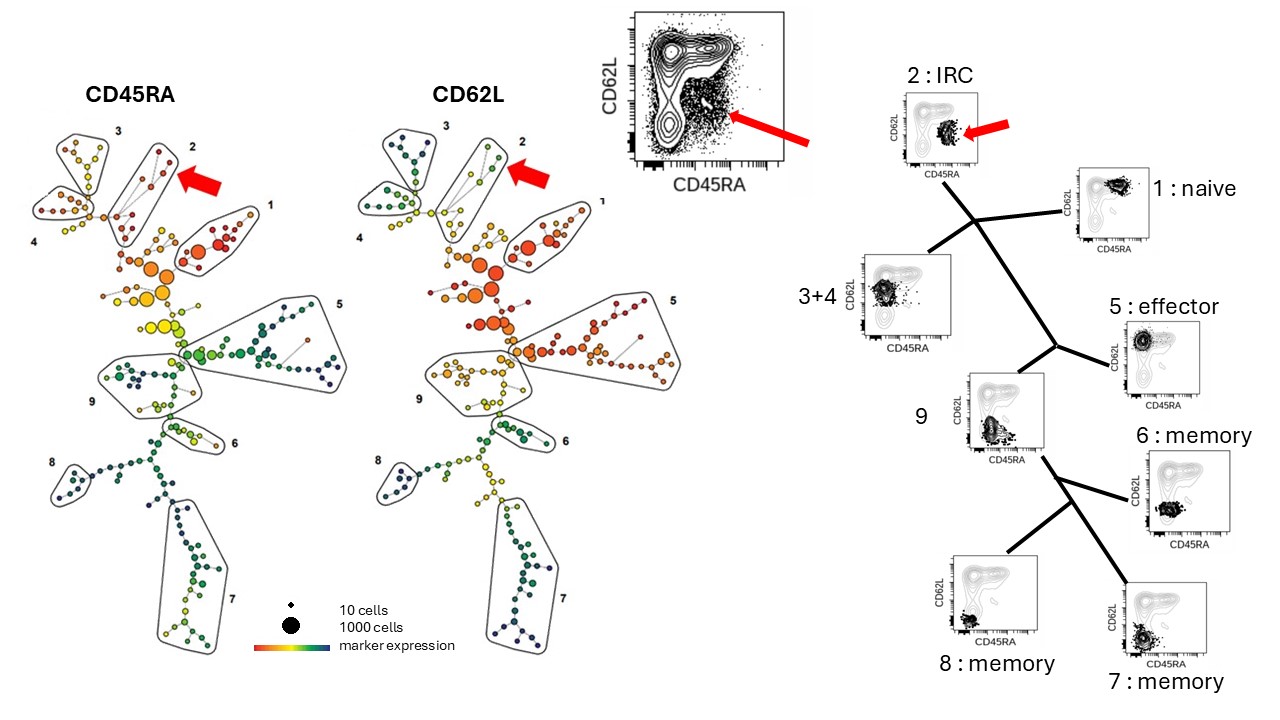


**Supplementary Figure S3: Healthy Controls**

**Left panels :** Age relationship in healthy controls were established for naïve and Treg CD4+T. IRC were not related to age.

**Right panels** : Data distribution for Naïve and Treg frequencies after normalisation.

**
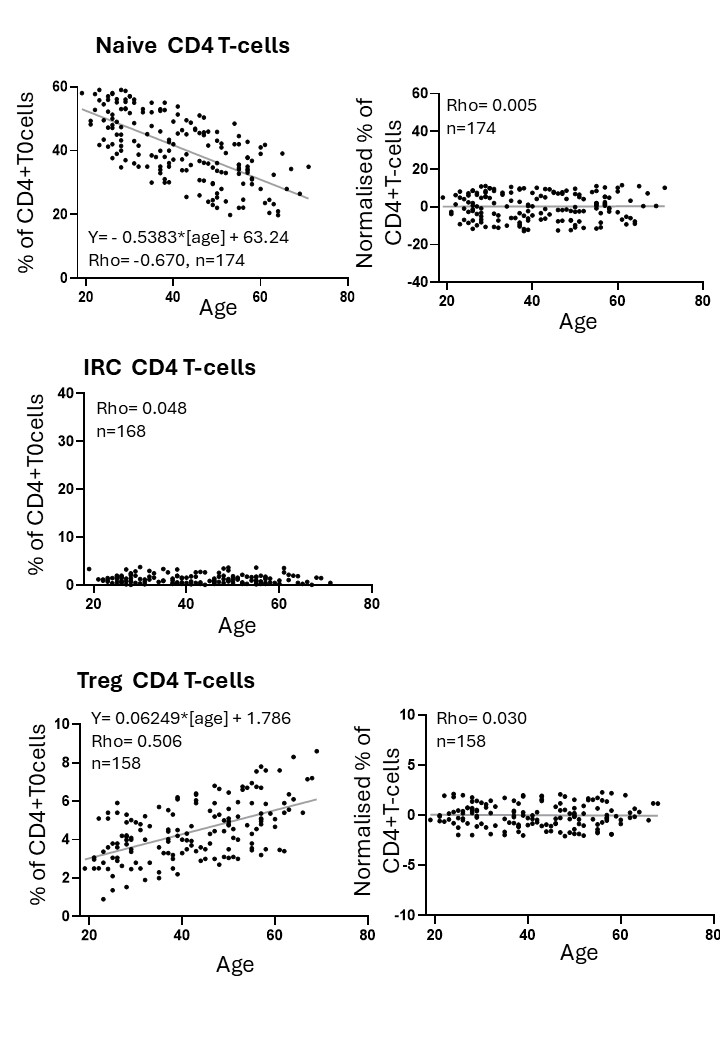
**

**Flow-cytometry data quality and acceptability**

#### Problems encountered

*Poor blood quality*

This can impact any test performed routinely on blood. An acceptable quality of sample for optimal flow cytometry is presented (see figure below) that allows clear identification of cell population of interest. In contrast poor red cell lysis RCL) resulting from biological characterisation such as high viscosity, inflammation, is impacting the ability of fluorochrome to work optimally (see details in plot below). RA having high inflammatory markers in the blood, this was to be expected. As such, we observed cases where RCL was far from optimal, and the sample was almost impossible to read. This affects both the FSC/SSC and marker detection. It should not cause major issues as the RCL can be repeated as is often the case in research settings and simple visual inspection often is sufficient to ascertain when needed.

*Delayed processing*.

In some cases, marker expression was “blurred”, (impossible to gate) but difficult to explain as RCL was efficient. It was noticed that this was usually in sample delayed in transit from clinics to the lab, via transport services (≥24 hours) which are likely to impact the test ^[8]^.

*Faulty processing*

This involved non-adherence to sample preparation protocol or omission of an antibody. The non-availability of certain antibodies in a panel explains most cases. This impacted mainly the Treg panel due to missing antibodies (FoxP3 or CD127) but sometime seen also in other panels as well (CD4) which fully prevented analysis. Results stemming from this “human” error occurred mostly in the Treg panel due to antibody shortage. High level of multiple pipetting procedure (particularly in the Treg panel with surface and intracellular staining) may explain in part this observation although it is hard to explain why a panel whose gating depends on a particular marker (FoxP3, CD4) can be performed when such antibody is not added. The second source of error in the Treg panel was the absence of CD127; which leads to over-estimation Treg frequency. Although it may have a limited effect on data according to other studies where CD127 was not used ^[9]^, it is a serious departure from the SOP defining the markers required to characterised human Treg cells ^[10]^. This should be added to the data report provided to clinicians to make decision. It appeared that CD4 omission were genuine pipetting mistake

*Instrument set-up*:

Another issue was the lack of adjustment of instrument settings on a daily basis**,** which can be performed to rescue on poor quality samples in research setting. This is an issue observed mainly in routine analysis as protocol are set “in stone”, settings being fixed and not allowing any adjustment. NHS data acquisition uses fully compensated protocols as standards. It appears that some protocols were updated over the years as some commercial antibody/dies were replaced and also every time the software upgraded automatically. Data were then acquired with un-used channels which caused a shift in compensation and went un-noticed by the operators. This is a technical hurdle that can be overcome but at the cost of a highly time-consuming process and wrong data reports that needed to be corrected at posteriori.

**Example of FCS plots illustrating problem encountered during analysis.**

- Dual plot of size (FSC) versus granularity (SSC) of good (left) versus poor-quality of blood, most cell lysed (middle panel), or inefficient red cell lysis (right panel).
- Plot showing missing staining by anti CD127 and FoxP3 (black circle) compared to plot representing the Treg gate (blue circle).
- Example of sample showing uncompensated plot in 2 panel (top) followed by the same plot re-compensated (bottom).


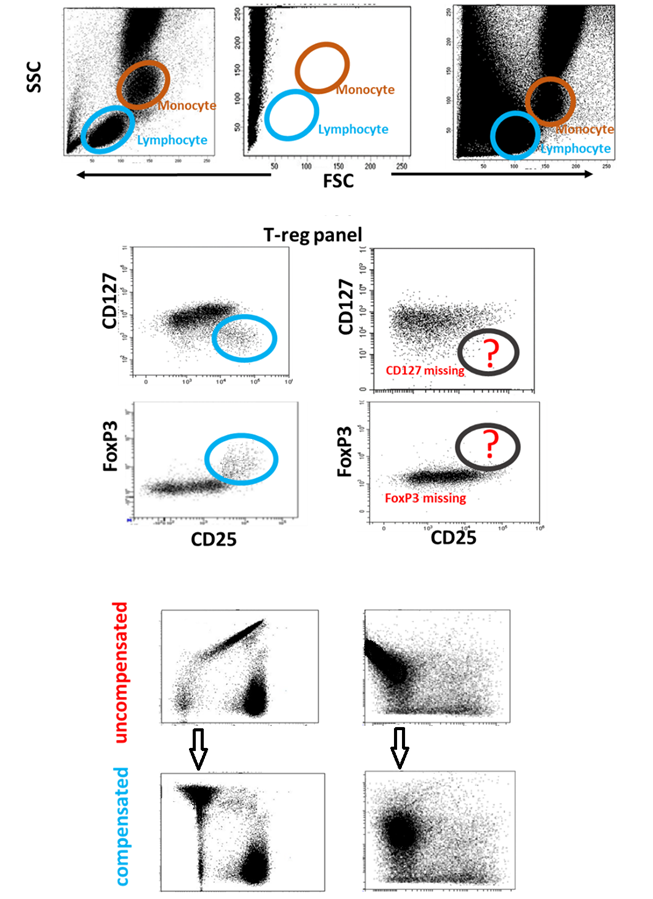


Based on these observations, we detected 3 types of error requiring the exclusion of the samples with unacceptable data quality, considering human error, technical issues, and poor sample quality. Transport leading to poor quality affected more the T-cell panel (Naïve, ICR) most likely because the CD62L markers is known to be sensitive (pre-analytical conditions, temperature, notably storing blood at 4^o^C compromises CD62L detection greatly). Human error which included doing the test when Ab are missing affected more the Treg panel, while machine/software issues affected both. Altogether >10% of samples should have been excluded (not reported upon) for naïve/IRC of which over half could be rescued by recompensation. For Treg, all the test with no antibodies should not have been carried out as nothing can be performed at posteriori to rescue the data (with financial issue).

**Exclusion of flow cytometry raw data files based on faulty acquisition.**

Each pie chart represented the results of excluding samples based on “processing” error (blue), technical error (green), and poor blood quality (red). Acceptable samples (passed QA/QC) are represented in grey colour.


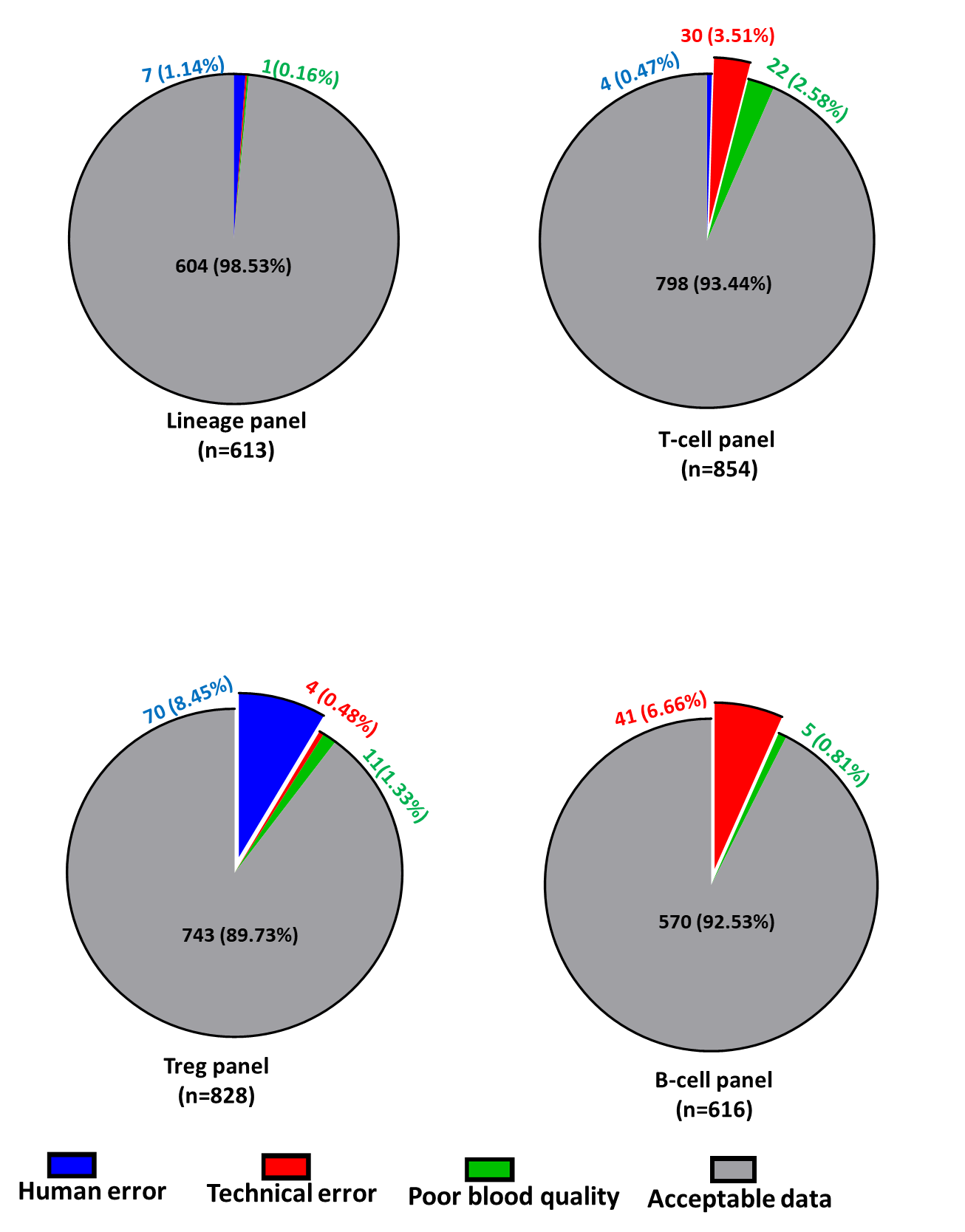

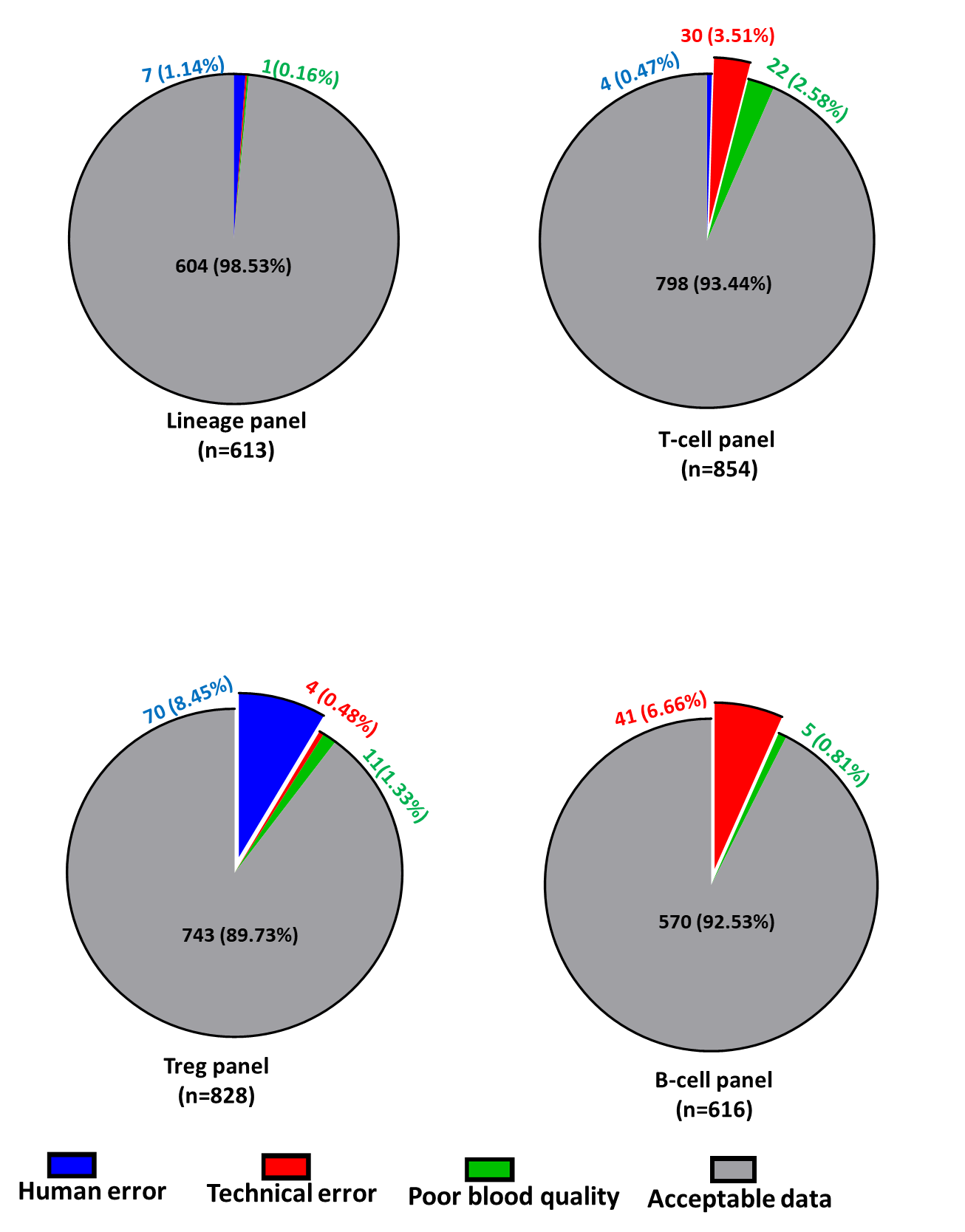


This led to amendment to the protocols with tests no longer being performed for panel when

- essential antibodies where not available (CD4, FoxP3)
- delays in transport of > 24 hours
- disabling of some automatic software update / annual maintenance visits
- additional planning for reagent management
- additional step for RCL were not adopted

**Performance evaluation of inter-observers' variability in gating**

Flow cytometry raw data from the T-cell and Treg panels were routinely reported on NHS servers (NHS-staff). The SOPs used by the NHS including the gating processes originally designed when the protocol was transferred from our research lab in 2013**.** The NHS-reported results were made available and Raw data were re-analysed independently (research staff IAC) and then utilized to determine the inter-observers' variability in the cell subset frequencies reported. IA was first trained in flow cytometry by FP (>20 years of experience in flow), who developed those panels in research settings and transferred them to hospital services. To validate the reproducibility of data between observers one was considered the reference (IA) and the other (NHS) the challenger.

Paired samples comparing results from both observers included: n=262 for naïve CD4+T cells, n=203 for IRC CD4+T cells, and n=162 for Treg CD4+T cells (from one cohort over the period 2015-2019). Data were excluded from any samples presenting issues as defined above.

NHS results being acquired over 10 years, were divided into 3-time period corresponding to the development of the service with staff-1 (2013-2014), 2^nd^ to the routine use of the service with staff-2 (2015-2019), and 3^rd^ over COVID pandemic with staff-3 (2020-2021 over the pandemic). The general characteristics of the distribution of data for the 3 biomarkers (Naive, IRC, Treg) were evaluated using paired t-test. For Naïve, the mean of difference between the two observers did not differ significantly first [0.17% (95%CI -0.65 to 0.98), p=0.681) and middle period [1.249 (0.0265 to 2.471)] in contrast to the last periods [4.39% (1.309 to 2.897)], suggesting a loss of data consistency over 2020-21. For IRC, data distribution across the 3 periods of time did not differ significantly (all p>0.05), suggesting a better consistency in overall gating pattern for IRC subsets, although with some outliers. For Treg, NHS2013-15 results were not significantly different between observers during the development/use of the service, while during the late period, data were less closely comparable. An analysis of agreement between observers was performed first using spearman correlation analysis and then Bland-Altman (BA) plots.

Correlations results are displayed using scatter plots with lines of best-fit to address the match between observers (correlation coefficients rho indicating the strength of the linear relationship) compared the line of equality which would suggest an absolute correlation between datapoints. For naïve CD4+T-cells, there was a highly significant correlation (rho=0.9336, p<0.0001) and both the line of best fit and the line of equality were closely overlapped. For IRC CD4+T, the correlation was still significant but with a decline in strength (rho=0.756, p<0.001). The line of best fit showed divergence from the line of equality, with particularly poor linearity of the relationship between observers when frequencies were low (<2-3%) while the high frequencies were actually driving the correlation. This suggests a poor consensus between observers. For Treg, the correlation coefficient reduces even further (rho=0.688) while lines of best-fit and equality were close. Two points were particularly far apart suggesting a possible additional sources of error in data entry reported on server (9% and 10% frequencies instead of 0.9% and 1%).

We further assess level of the agreement between observers using Bland-Altman (BA) plots, constructed for each subset by plotting the difference between measurements from the two observers (challenger - reference) against the average of both observers. If no difference were to be seen, the mean of those differences (green) should be equal to zero (black line) and the 95% CI should be small (dotted red lines) and indicative of the error in measurement. For naïve cells, the result of the measurement bias between observers was –2% (mean differences) which is close to zero compared to naïve cells frequencies usually >30%. The BA plot suggests a range of the difference between observers with a few outliers (outside 95% CI red lines, poor blood quality). The hypothesis that the observed mean of the difference (-1.9%) was not different from zero (= no bias between observers) was tested (t-test of the difference values, p=0.01) which suggests a certain degree of measurement bias between the 2 observers, with most result by the challenger consistently under-evaluated compared to reference (by a mean of 2%). For IRC, the mean difference (green line) was superimposed on the assumed mean difference of zero (black line) with little or no suggestion of bias (T-test p=0.400). With the BA plot showing a range in measurement difference (-2.35 to +2.36%) between observers that is often within the range of most frequencies of IRC themselves, it suggests that IRC results when below 2.5% should be considered “noise” in measurements, while higher frequencies should be considered abnormal/raised frequencies. For Treg, the bias was –0.4% (green line), but the test of evidence of bias (p<0.001), suggested again that most results by the challenger were consistently under-evaluated compared to reference.

This BA analysis therefore suggests the possibility of error between measurements between observers. Intraclass correlation coefficient (ICC), is also recommended as an index for reliability test between 2 measurements, as it reflects not only the degree of correlation (i.e., the strength of linear relationship) but also the agreements between 2 measurements ^[11]^. High reliability or low measurements error should be equal (or close) to a score of 1 whereas low reliability or high measurements error will show a score close to null. ICC values above 0.9 are considered of excellent reliability between 2 measures ^[12]^. The average ICC between challenger/reference was quite high for naïve cells [(0.963, 95%Cl 0.937-0.977)], indicating good reliability between both observers. For IRC [0.776, 0.705-0.830] and Treg [0.781, 0.678-0.848], scores were lower confirming some level of discrepancy in data reported by observers and the need for more experienced staff over the analysis of data.

The involvement of different staff (3 challengers) over 10 years may have played an important role in impacting the gating consistency considering the subjectivity of the process and the need for training and experience in staff (research or routine) when performing flow. Nonetheless, protocols are transferable from research to hospital services and flow is commonly used to make clinical decisions, suggesting that these biomarkers can be used widely.

**Left: Comparison between frequencies identified 2 observers (Spearman correlation analysis).** Data displayed in scatter plot with Line of best fit (dotted line, slope), line of equality (blue line, slope=1) and rho coefficient of correlation for the 3 subsets. Intraclass correlation coefficient (ICC) indicates the reliability results between observers.

**Right: Bland-Altman plots of the CD4+T cells subset quantification comparing the challenger and reference.** Data displayed in BA plot with 95% CI of the range of results between observers (dotted red line), expected mean difference (dark line at zero), observed mean difference (green line).


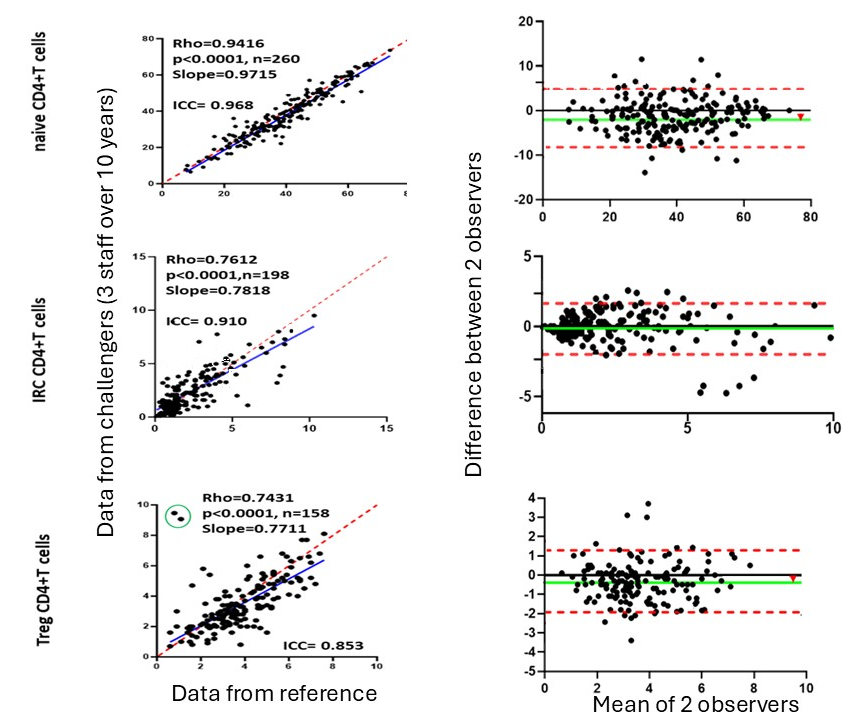


**Detail of modelling analysis for outcome prediction across the IAC**

***Methodology***

ROC analysis were performed to establish individual predictive values were relevant. Sensitivity as well as odds ratio (OR) and positive and negative predictive values (PPV/NPV) were calculated.

For multivariate analysis, binary logistic regression models were constructed for the prediction of outcomes following missing data imputation performed for variable with <10% missing data at random confirmed by Little’s MCAR test. Missing data imputation was performed using the SPSS tool for multiple imputation allowing boundaries of data distribution to be inputted over five cycles. The estimates obtained from 5 dataset were aggregated to produce an overall imputation estimate. The pooled dataset was compared with the non-imputed dataset to verify that ORs were not affected by the imputation process, which were not. We used a forward regression approach allowing the sequential selection of the best predictors.

***Dichotomisation***

To use of T-cell biomarkers practically, we determined Cut-off for 3 main outcomes, defining high/low risk, and examined the overlap between them (Venn diagram below). Thresholds for dichotomisation were set at ~80% specificity (for individual outcomes) as high and low risk for an outcome.

Dichotomisation suggested 30% of patients had **high-risk of progression** with naïve cells (cut-off <-6.5% naïve), 39% for IRC (>2.5%) and 35% for Treg (<-1.4%) showed complementarity between the biomarkers with 28/442 (6.5%) having all 3 biomarkers, 98 (22%) with 2 biomarker and 156 (35%) with only 1. Over annual-repeats in Pr, a switch from low- to high-risk was observed for naive (31/57 (54%), while conversely, only 1 patient switch from high- to low-risk. In the NP, 39/55 (70%) of annual repeats were in the low-risk group and all but 1 stayed there with no change in the high-risk group. Similar observations were obtained for IRC (cut-off >+2.5%) with 16 patients switching from low- to high-risk over time and 12 patients for Treg (cut-off <-1.4%).

**For classification**, 270/374 (72%) were predicted by naïve (risk cut-off <10%), and 307/374 (82%) by Treg (cut-off -1.5%), but not as well by IRC (93/374 (26%). There was large overlap between naïve and Treg high-risk, while all but 3/141 of the high-risk IRC were not associated with another T-cell biomarker. This was reproduced for ACPA- participants with only 7/69 (10%) RA patients not being associated with either high-risk for naïve or Treg, 3/7 being high-risk IRC.

**For predicting MTX-induced remission** only naïve cell were predictive (>2.5%) and 85/126 (67.5%) of the high-chance group did achieve remission while 20/98 (20.5%) in the low-risk category also achieved remission.


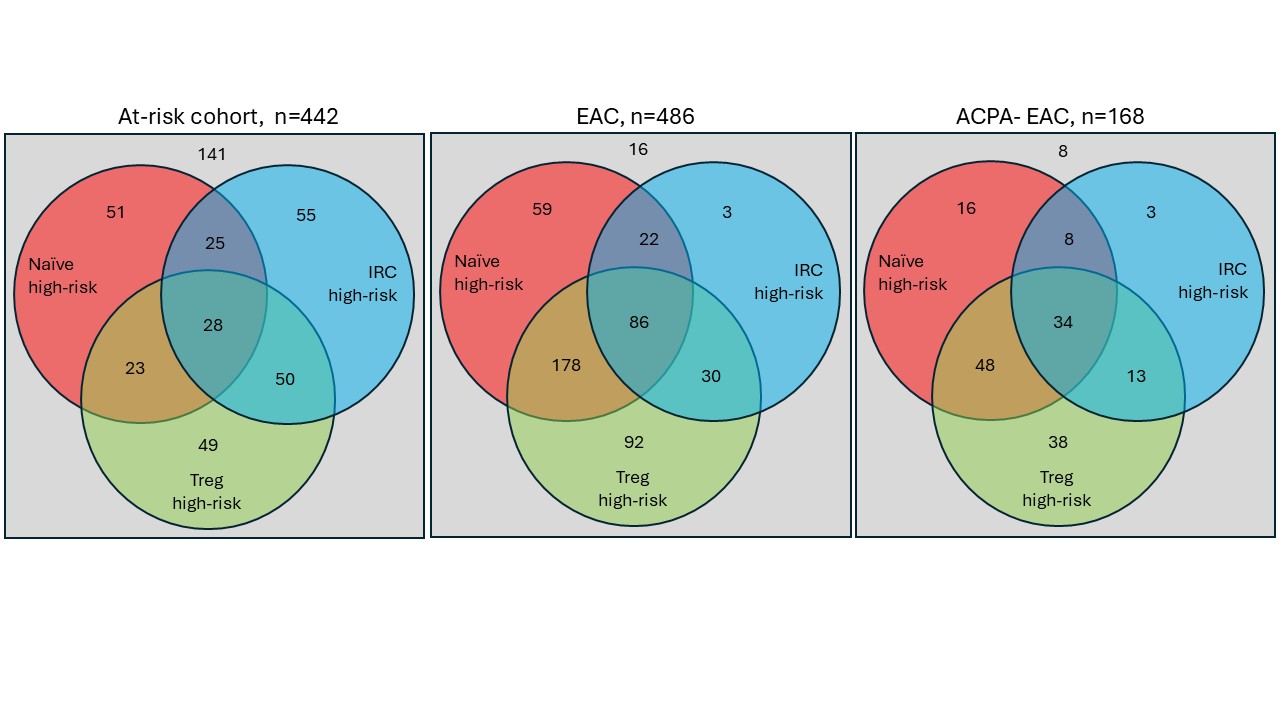


***Progression in at risk individuals:***

Validation of our previous model predicting progression to IA using the 3 bimarkers is presented in supplementary material (SUPP-Table-S1, n=442). Comparing clinical data alone and clinical+T-cell data, a gain in +8% of the model accuracy (77.1%) and +0.100 AUC (AUC=0.848) was observed between models allowing for selecting the best predictors (Forward method). Of note, a model including only the 3x CD4+T-cells subsets (73.5% accuracy, AUC=0.798) was already more accurate than models including the clinical data alone (69.5% accuracy, AUC=0.748). Dichotomisation of data for a high risk of progression for the 3 T-cells subsets (using >80% specificity in individual binary logistics regressions) suggested cut-off at <-6.5% or normalised Naïve, >2.0% for IRC and <-1.4% for Treg. Combining the 3 subsets into a 0->3 T-cell risk score (by adding individual dichotomised risk high=1, low=0), this score was associated with progression (p=1.9 x10^-22^, OR=3.630, AUC=0.770) but was not as good (74.4% accuracy AUC=0.822) as the model combining clinical+T-cell data, while still better that clinical data alone.

***Early RA classification***

A logistic regression model (SUPP-Table-S2, excluding SE and GH due to high missingness) for RA classification according to EULAR 2010 criteria suggested a modest gain of +2% accuracy and +0.010 AUC using the T-cell subsets over the clinical only data (n=486).

We repeated the analysis of the added value of T-cell subsets, whereby patients >60 were excluded. Despite losing 113 RA and 5 non-RA (n=373), we still observed a +2% gain in accuracy and a better gain in AUC (+0.020).

ACPA-negative patients (RA n=61 and non-RA n=97) are in need alternative classification biomarkers due to the reliance on ACPA for RA classification. In this group, T-cell subset (naïve and IRC, not Treg) provided a substantial accuracy gain in of +4.5% and +0.042 AUC.

***Early RA response to MTX***

We previously demonstrated the value of naïve CD4+T-cells in predicting MTX-induced remission (2014-2020). Here we used a different modelling approach to demonstrate the added value of naïve subset over clinical data only in a forward approach allowing the selection of the best predictors. We combined MTX treated patients from the EAC register and the control arms of 2 clinical trials, allowing for 221 patients to be used (Figure-3a). Naïve (but not IRC and Treg) were highly predictive of MTX-induced remission (p=4.6x10^-5^). We performed modelling of clinical data alone (SUPP-Table-S3, accuracy=68% AUC=0.731),

then adding naïve T-cell subsets (accuracy=70.4%, AUC=0.765) and confirmed the added value of using T-cells (+4.4% accuracy). Most gain was seen in +6% sensitivity, +5% PPV and + 4% NPV.

Supplementary Table S1: Characteristics of ACPA+ arthralgia at-risk individuals and model predicting progression to IA (n=442)

|  | **Unadjusted** | | | | | | **FORWARD method** | |
| --- | --- | --- | --- | --- | --- | --- | --- | --- |
|  | Missing | Prog  (n=198) | Non-Prog  (n=244) | p-value | OR  (95% CI)  p-value | AUC*  (95%CI)  p-value | Clinical  only | Clinical +  T-cell subsets |
| ^$^Age | 0 | 53  (19)  [20 to 82] | 50  (18)  [18 to 82] | 0.092 | 1.0  (1.0 to 1.0)  0.0321 | 0.527  (0.466 to 0.588)  0.394 | Not  selected | Not  selected |
| Sex  Male(%)  Females(%) | 0 | 67 (33.9%)/  131 (66.1%) | 64 (26.2%)/  180 (73.8%) | 0.091 | 0.8  (0.5 to 1.1)  0.0839 | 0.461  (0.403 to 0.516)  0.172 | Not  selected | Not  selected |
| ^$^ Duration of Arthralgia (weeks) | 34 | 14  (33)  [0 to 457] | 16.5  (28)  [1 to 445] | 0.299 | 1.001  (0.997 to 1.004) 0.759 | 0.469  (0.410 to 0.528)  0.289 | Not  selected | Not  selected |
| RF  Neg(%)  Pos(%) | 12 | 77 (40.5)  113 (59.5) | 169 (71.9)/  66 (28.1) | 7.6 x10^-11^ | 3.75  (2.502 to 5.640)  1.6 x10^-10^ | 0.657  (0.604 to 0.710) 2.6x10^-8^ | 3.367  (2.196 to 5.171)  2.9 x 10^-8^ | 3.351  (2.056 to 5.462)  1.2 x 10^-6^ |
| CCP-2 levels | 9 | 105  (280)  [2 to 340] | 14  (82)  [2 to 340] | 7 x10^-11^ | 1.004  (1.003 to 1.006)  1.3 x10^-8^ | 0.7  (0.6 to 0.7)  1.5 x10^-13^ | * | ** |
| ^#^ HLA-DR  Neg(%)  Pos(%) | 39 | 48 (29.1)  117 (70.9) | 128 (54.9)  105 (45.1) | 3.1 X10^-7^ | 2.971  (1.945 to 4.540)  4.6 X10^-7^ | 0.6  (0.6 to 0.7)  0.0006 | 2.917  (1.918 to 4.621)  1.1 x 10^-6^ | 2.772  (1.687 to 4.557)  5.7 x 10^-5^ |
| ^#^Smoking  Never(%)  Ever(%)_ | 2 | 62 (31.3)  136 (68.7) | 120 (49.6)  122 (50.4) | 8.4 x10^-5^ | 2.181  (1.469 to 3.236)  1.2 x10^-4^ | 0.596  (0.539 to 0.646)  0.001 | Not  selected | Not  selected |
| ^$^EMS(mins) | 3 | 10  (45)  [0 to 480] | 2  (30)  [0 to 560] | 0.002 | 1.003  (1. to 1.005) 0.061 | 0.583  (0.530 to 0.637)  0.003 | Not  selected | Not  selected |
| ^$^ESR | 13 | 13  (18)  [1 to 82] | 10  (13)  [1 to 74] | 0.002 | 1.026  (1.009 to 1.044)  0.002 | 0.589  (0.535 to 0.644)  0.002 | Not  selected | Not  selected |
| TJC-78 | 0 | 2  (4)  [0 to 27] | 0  (3)  [0 to 16] | 0.004 | 1.072  (1.009 to 1.140)  0.025 | 0.577  (0.523 to 0.631)  0.006 | 1.072  (1.002 to 1.146)  0.042 | Not  selected |
| ^$^CRP  (high sensitivity) | 23 | 5.3  (7.8)  [0 to 66] | 1.2  (5.5)  [0 to 26] | 8.8 x10^-6^ | 1.071  (1.034 to 1.110)  0.00014 | 0.626  (0.567 to 0.685)  5.5 x10^-5^ | 1.055  (1.018 to 1.093) 0.003 | 1.053  (1.011 to 1.097)  0.012 |
| ^$^Naive  T-cells | 18 | -2.9  (21)  [-27 to 33] | +4.8  (20)  [-23 to 39] | 3.5x10^-7^ | 0.959  (0.944 to 0.975)  3.4 x10^-7^ | 0.354  (0.296 to 0.412)  3.2 x10^-7^ |  | 0.951  (0.932 to 0.970)  1.3 x 10^-7^ |
| ^$^IRC | 18 | 2.3  (3.6)  [0 to 36] | 1.0  (1.7)  [0 to 10.6] | 4.3 x10^-9^ | 1.275  (1.160 to 1.399)  2.8x10^-7^ | 0.655  (0.599 to 0.713)  7.8x10^-7^ |  | 1.148  (1.029 to 1.282)  0.013 |
| ^$^Treg | 64 | -1.4  (2.4)  [-5.5 to 3.5] | -0.2  (2.5)  [-4.7 to 5.9] | 1.7 x10^-13^ | 0.623  (0.543 to 0.712)  9.1x10^-12^ | 0.272  (0.219 to 0.326)  3.2 x10^-13^ |  | 0.582  (0.496 to 0.683)  3.6 x 10^-11^ |
| Accuracy  (%) |  | | | | | | 69.5  (66.27 to 75.00) | 77.5  (72.95 to 81.02) |
| AUC |  |  |  |  |  |  | 0.749  (0.703 to 0.795)  8.2 x 10^-19^ | 0.851  (0.812 to 885)  4.4 x 10^-35^ |
| SEN (%) |  |  |  |  |  |  | 70  (62 to 77) | 78  (71 to 84) |
| SPE (%) |  |  |  |  |  |  | 70  (65.5 to 76.5) | 79  (71 to 81.5) |
| PPV (%) |  |  |  |  |  |  | 59  (55 to 65) | 70  (62 to 77) |
| NPV (%) |  |  |  |  |  |  | 80  (75 to 8) | 85  (81 to 88) |

^$^Numerical variables reported as median (IQR) [min to max]; #Categorical variables reported as number and frequency (%) of patients. HLA (SE) human leucocyte antigen (shared epitope); TJC tender joint count; RF rheumatoid factor; Treg regulatory T-cells; IRC inflammatory-related cells;^$^ normalised frequency OR odd ratio; AUC area under the roc curve (95% CI) and p-value. SEN/SPE sensitivity/specificity PPV/NPV positive/negative predictive value. CI Confidence interval. * Adding levels of ACPA (2^nd^ Gen test) improved by +0.8% accuracy and +0.08 AUC in the clinical only model but had no additional effect on the Clinical +T-cell model (+0.0% accuracy, +0.002 AUC).

Tests adjustment for multiple comparisons (adjustment of the *P-value*) was performed by applying Bonferroni correction method for multiple comparison test).

Supplementary Table S2: Characteristics and modelling for RA classification in an early inflammatory arthritis clinic cohort (n=417).

|  | | **Unadjusted (n=417)** | | | | | **All participants**  OR (95% CI) p-value | | | | | **ACPA-negative participants (n=168)** OR (95% CI) p-value | |  |
| --- | --- | --- | --- | --- | --- | --- | --- | --- | --- | --- | --- | --- | --- | --- |
|  | missing | RA  (n=302) | Non-RA  (n=115) | p-value | OR  (95% CI)  p-value | AUC*  (95%CI)  p-value | Clinical only | Clinical  + T-cells | | | Clinical only | | Clinical  + T-cells |  |
| ^$^Age | 0 | 51  (19)  [21 to 87] | 48  (22)  [19 to 90] | 0.006 | 1.032  (1.015 to 1.048),  0.00014 | 0.566  (0.56 to 0.68)  0.0006 | 1.026  (1.006 to 1.005)  0.014 | 1.027  (1.002 to 1.052)  0.032 | | | 1.035  (1.01 to 1.061)  0.010 | | 1.041  (1.013 to 1.070)  0.005 |  |
| ^#^ Male  Females | 0 | 84 (28.0)  218 (72.0) | 42 (37.5)  73 (63.5) | 0.064 | 1.6  (1.00 to 2.49),  0.152 | 0.536  (0.41 to 0.61)  0.531 | Not selected | Not selected | | | Not selected | | Not selected |  |
| ^$^Symptoms Duration  months | 66 | 5  (5)  [0 to 26] | 6  (10)  [1 to 24] | 0.046 | 0.950  (0.912 to 0.990),  0.013 | 0.387  (0.30 to 0.46)  0.005 | Not selected | Not selected | | | Not selected | | Not selected |  |
| ^#^RF  neg  pos | 33 | 97 (35.0) 180 (65.0) | 91 (85.0)  16 (15.0) | 4.0  x10-^18^ | 14  (7.5 to 26),  3.2 x 10^-15^ | 0.731  (0.62 to 0.84)  7.0 x10^-15^ | 3.044  (1.392 to 5.907)  0.002 | 2.873  (1.352 to 6.101)  0.010 | | | 6.525  (2.158 to 18)  0.00037 | | 5.487  (1.700 to 15.32)  0.002 |  |
| ^#^ACPA  neg  pos | 15 | 71 (24.4) 224 (75.6) | 97 (84.4)  10 (15.6) | 6.8  x10^-31^ | 40  (20 to 81),  8.4 x10^-24^ | 0.860  (0.70 to 0.92)  4.0 x10^-28^ | 21  (10 to 44)  9.3 x10^-16^ | 24.5  (12 to 52)  2.3 x10^-16^ | | |  | |  |  |
| ^#^Smoking never  ever | 60 | 109 (40.2)  163 (59.8) | 36(42.4)  49(57.6) | 0.823 | 1.227  (0.66 to 1.80),  0.725 | 0.525  (0.44 to 0.60)  0.765 | Not selected | Not selected | | | Not selected | | Not selected |  |
| ^$^TJC-28 | 32 | 8  (10)  [0 to 28] | 4  (10)  [0 to 44] | 2.4  x10^-3^ | 1.037  (1 to 1.074),  0.0474 | 0.624  (0.55 to 0.72)  0.0002 | Not Selected | Not Selected | | | Not selected | | Not selected |  |
| ^$^SJC-28 | 32 | 5  (7)  [0 to 28] | 1  (4)  [0 to 12] | 3.9  x10^-9^ | 1.220  (1.124 to 1.325),  6.6 x10^-8^ | 0.704  (0.64 to 0.76)  2 x10^-9^ | 1.149  (1.074 to 1.230)  0.000087 | 1.167  (1.056 to 1.218)  0.000035 | | | 1.124  (1.03 to 1.184)  0.001 | | 1.148  (1.060 to 1.241)  0.00035 |  |
| ^$^CRP | 67 | 8  (24)  [0 to 228] | 4  (11)  [0 to 163] | 1.7  x10^-4^ | 1.013  (1.001 to 1.026),  0.038 | 0.651  (0.58 to 0.72),  1.5 x10^-5^ | Not selected | Not selected | | | Not selected | | Not selected |  |
| ^$^Naive T-cells | 0 | -1.6  (21)  [-36 to 32] | 5.6  (20)  [-23 to 40] | 0.0009 | 0.980  (0.95 to 0.98),  0.0007 | 0.418  (0.36 to 0.48),  0.009 |  | 0.969  (0.950 to 0.994)  0.007 | | |  | | 0.974  (0.945 to 0.997)  0.029 |  |
| ^$^IRC | 18 | 2.8  (3)  [0 to 33] | 3.1  (9)  [0 to 38] | 0.118 | 0.932  (0.903 to 0.963),  0.00005 | 0.450  (0.37 to 0.53),  0.118 |  | 0.909  (0.865 to 0.956)  0.00025 | | |  | | 0.898  (0.840 to 0.960)  0.002 |  |
| ^$^Treg | 89 | -.0.4  (3)  [-6 to 8] | -1.3  (4)  [-6 to 7] | 0.011 | 0.888  (0.81 to 1.090),  0.071 | 0.452  (0.41 to 0.68),  0.107 |  | 0.827  (0.726 to 0.941)  0.004 | | |  | | Not selected |  |
| Accuracy  % |  | | | | | | 86.4 | | 88.4 | 69.3 | | | 75.6 | |
| AUC,  95% CI  p-value |  |  |  |  |  |  | 0.904  (0.882 to 0.946)  2 x10^-36^ | | 0.917  (0.88 to 0.94)  4.3 x10^-39^ | 0.869  (0.772 to 0.921)  6.3 x10^-6^ | | | 0.882  (0.860 to 0.995)  2.8 x10^-6^ | |
| SEN |  |  |  |  |  |  | 92.  (89 to 94) | | 92.5  (90 to 95) | 67  (53 to 79) | | | 73.5  (60 to 84) | |
| SPE |  |  |  |  |  |  | 71  (62 to 79) | | 75  (66 to 82) | 71  (62 to 78) | | | 77  (67 to 84) | |
| PPV |  |  |  |  |  |  | 91  (89 to 94) | | 93  (90 to 94) | 51  (42 to 59) | | | 64  (55 to 72) | |
| NPV |  |  |  |  |  |  | 74  (66 to 80) | | 74  (66 to 81) | 83  (76 to 88) | | | 84  (77 to 89) | |

|  | | **RA < 59 years of age (n=304)**  OR (95% CI) p-value | | **RA > 60 years of age (n=143)**  OR (95% CI) p-value | | | |  |
| --- | --- | --- | --- | --- | --- | --- | --- | --- |
|  | Clinical only | | Clinical  + T-cells | | Clinical only | | Clinical  + T-cells | |
| ^$^Age | Not selected | | Not selected | | Not selected | | No  value  for  the  model | |
| ^#^ Male  Females | 2.256  (1.060 to 4.55)  0.035 | | Not selected | | Not selected | |  |  |
| ^$^Symptoms Duration  months | Not selected | | Not selected | | Not selected | |  |  |
| ^#^RF  neg  pos | 2.636  (1.1 to 5.599)  0.019 | | 2.436  (1.157 to 5.590)  0.045 | | Not selected | |  |  |
| ^#^ACPA  neg  pos | 27  (12 to 63)  7.3 x 10^-16^ | | 29  (13 to 69)  2.3 x 10^-14^ | | 38  (8 to 186)  5.3 x 10^-6^ | |  |  |
| ^#^Smoking never  ever | Not selected | | Not selected | | Not selected | |  |  |
| ^$^TJC-28 | Not selected | | Not selected | | Not selected | |  |  |
| ^$^SJC-28 | 1.135  (1.048 to 1.231)  0.002 | | 1.125  (1.420 to 1.215)  0.002 | | 1.216  (1.038 to 1.422)  0.015 | |  |  |
| ^$^CRP | Not selected | | Not selected | | Not selected | |  |  |
| ^$^Naive T-cells |  | | 0.956  (0.933 to 0.980) 0.036 | |  | |  |  |
| ^$^IRC |  | | 0.895  (0.844 to 0.945)  0.00019^5^ | |  | |  |  |
| ^$^Treg |  | | 0.791  (0.684 to 0.913)  0.001 | |  | |  |  |
| Accuracy  % | 86 | | 89 | 88 | |  | | |
| AUC,  95% CI  p-value | 0.910  (0.867 to 0.935)  7 x10^-35^ | | 0.925  (0.897 to 0.954)  4 x10^-38^ | 0.910  (0.857 to 0.961)  1 x10^-8^ | |  |  |  |
| SEN | 88  (83 to 92) | | 92.5  (88 to 95) | 92  (85 to 96) | |  |  |  |
| SPE | 82  (73 to 90) | | 82  (70 to 87) | 57  (29 to 82) | |  |  |  |
| PPV | 93  (90 to 95) | | 93  (90 to 94) | 95  (91 to 97) | |  |  |  |
| NPV | 71  (64 to 78) | | 79  (71 to 85) | 42  (26 to 60) | |  |  |  |

$Numerical variables reported as median (IQR)[min to max]; #Categorical variables reported as number and frequency (%) of patients. HLA (SE), human leucocyte antigen (shared epitope); TJC, tender joint count; RF, rheumatoid factor; Treg, regulatory T-cells; IRC, inflammatory-related cells; ^$^ normalised frequency, OR, odd ratio; AUC area under the roc curve, SEN, sensitivity, SPE, specificity, PPV/NPV, positive / negative predictive value. CI, Confidence interval. 11 patients with non-RA could not be included due to missing data. Tests adjustment for multiple comparisons was performed by applying Bonferroni correction method for multiple comparison test.

**Supplementary Table S3: Characteristics and predictive models of MTX-induced remission in early RA (n=221)**

|  | | Unadjusted | | | | | | Logistic regression  OR (95% CI) p-value | | |
| --- | --- | --- | --- | --- | --- | --- | --- | --- | --- | --- |
|  | missing | Remission (n=112) | No remission  (n=109) | p-value | OR  (95% CI)  p-value | AUC*  (95%CI)  p-value | Clinical only OR (95% CI)  p-value | | Clinical +T-cells OR (95% CI)  p-value |  |
| ^$^Age | 0 | 58  (20)  [24 to 87] | 55  (17)  [19 to 87] | 0.057 | 1.02  (0.99 to 1.03)  0.076 | 0.426  (0.35 to 0.50)  0.055 | 1.030  (1.008 to 1.052)  0.007 | | Not Selected |  |
| ^#^Gender  Male  Female | 0 | 38 (33.9)  74 (66.1) | 32 (29.4)  77 (70.6) | 0.558 | 0.81  (0.45 to 1.43)  0.466 | 0.52  (0.45 to 0.59)  0.557 | Not Selected | | Not Selected |  |
| ^$^Symptom Duration | 1 | 22  (28)  [3 to 105] | 25  (24)  [3 to 104] | 0.316 | 0.99  (0.98-1.01)  0.545 | 0.45  (0.38-0.53)  0.315 | Not Selected | | Not Selected |  |
| ^#^RF  neg  pos | 9 | 48 (44.9)  59 (55.1) | 42 (40)  63 (60) | 0.564 | 0.82  (0.47 -1.41)  0.474 | 0.48  (0.39-0.55)  0.398 | Not Selected | | Not Selected |  |
| ^#^ACPA  neg  pos | 2 | 45(40.5)  66(59.5) | 41(38.0)  67(62.0) | 0.696 | 0.89  (0.52-1.54)  0.696 | 0.49  (0.41-0.56)  0.742 | Not Selected | | Not Selected |  |
| ^#^Smoking  never  ever | 2 | 57 (50.9)  55 (49.1) | 35 (32.5)  72 (67.3) | 0.010 | 0.47  (0.27-0.81)  0.007 | 0.41  (0.33-0.48)  0.018 | 0.541  (0.365 to 0.802)  0.002 | | 0.616  (0.410 to 0.926)  0.020 |  |
| ^$^TJC | 0 | 7  (11)  [0 to 31]) | 10  (13)  [0 to28] | 0.008 | 0.96  (0.93-0.99)  0.023 | 0.39  (0.32-0.47)  0.007 | 0.956  (0.918 to 0.996)  0.033 | | 0.952  (0.913 to 0.994)  0.024 |  |
| ^$^SJC | 0 | 4  (6)  [0 to 21] | 6  (9)  [0 to 22] | 0.018 | 0.94  (0.88-0.98)  0.011 | 0.41  (0.33-0.48)  0.016 | Not Selected | | Not Selected |  |
| ^$^CRP | 5 | 8  (17)  [0 to 158] | 15  (29)  [0 to 228] | 8.7 x10^-4^ | 0.98  (0.97-0.99)  0.005 | 0.37  (0.29-0.46)  0.001 | 0.982  (0.970 to 0.994)  0.003 | | 0.982  (0.971 to 0.994)  0.002 |  |
| ^$^GH-VAS | 68 | 49  (45)  [0 to100] | 60  (40)  [2 to 100] | 1.9 x10^-4^ | 0.98  (0.97-0.99)  6.2 x10^-4^ | 0.33  (0.24-0.41)  6.0 x10^-5^ |  | |  |  |
| ^$^Normalised naive T-cells | 0 | +7.1  (15)  [-33 to +44] | -2.3  (24)  [-30 to +40] | 4.6 x10^-5^ | 1.04  (1.02-1.07)  2.4 x 10^-4^ | 0.66  (0.59-0.73)  2.3 x 10^-5^ |  | | 1.050  (1.030 to 1.080)  9.5 x 10^-6^ |  |
| ^$^IRC | 0 | 2.1  (3)  [0 to 33] | 2.3  (4)  [0 to28 | 0.709 | 0.99  (0.94-1.06)  0.892 | 0.52  (0.44-0.59) 0.710 |  | | Not Selected |  |
| ^$^Normalised Treg | 2 | -1.7  (2)  [-6 to +7] | -1.0  (2)  [-5 to +8] | 0.077 | 0.94  (0.82-1.07) 0.363 | 0.43  (0.35-0.51) 0.080 |  | | Not Selected |  |
| Accuracy (%) |  | | | | | | 61.6 | | 69 |  |
| AUC |  |  |  |  |  |  | 0.731  (0.663 to 0.799)  1.3 x10^-8^ | | 0.765  (0.670 to 0.831)  7.2 x10^-11^ |  |
| SEN  SPE  PPV  NPC |  |  |  |  |  |  | 61  (52 to 69)  62  (51 to 73)  70.5  (64 to 77)  52  (45 to 59) | | 70  (60 to 77)  68  (58 to 78)  73  (65 to 78)  65  (57 to 72) |  |

$Numerical variables reported as median (IQR)[min to max]; #Categorical variables reported as number and frequency (%) of patients. HLA (SE), human leucocyte antigen (shared epitope); TJC, tender joint count; RF, rheumatoid factor; Treg, regulatory T-cells; IRC, inflammatory-related cells; ^$^ normalised frequency, OR, odd ratio; AUC area under the roc curve, SEN, sensitivity, SPE, specificity, PPV/NPV, positive / negative predictive value. CI, Confidence interval. Tests adjustment for multiple comparisons was performed by applying the Bonferroni correction method for multiple comparison test.

Supplementary Table S4: Characteristics and lack of association with TNFi-induced remission (n=78).

|  | | Unadjusted values | | | | | |
| --- | --- | --- | --- | --- | --- | --- | --- |
|  | missing | Remission  (n=48) | No remission  (n=30) | p-value | OR  (95% CI)  p-value | AUC*  (95%CI)  p-value |  |
| ^$^Age | 0 | 50.  (20.5)  (25 to 80) | 48.5  (18.2)  (31 to 72) | 0.817 | 0.995  (0.960 to 1.032) 0.797 | 0.484  (0.356 to 0.613)  0.812 |  |
| ^#^ Male  Females | 1 | 15(31.9)  32(68.1) | 10(33.3)  20(66.7) | 0.545 | 1.067  (0.402 to 2.830)  0.897 | 0.507  (0.374 to 0.640) 0.917 |  |
| ^$^Symptoms Duration | 18 | 16.8  (15)  (3 to 49) | 21.5  (37)  (7.5 to 44.5) | 0.548 | 0.986  (0.943 to 1.032) 0.544 | 0.454  (0.303 to 0.605) 0.547 |  |
| ^#^RF neg/positive | 1 | 14(29.8)  33(70.2) | 11(36.7)  19(63.3) | 0.350 | 1.365  (0.517 to 3.602) 0.530 | 0.534  (0.401 to 0.668) 0.613 |  |
| ^#^ACPA neg/positive | 1 | 7(14.9)  40(85.1) | 8(26.7)  22(73.3) | 0.164 | 2.078  (0.665 to 6.497) 0.209 | 0.559  (0.425 to 0.693) 0.390 |  |
| ^#^Smoking Status never/ever | 0 | 21 (43.8)  27 (56.3) | 14(46.7)  16(53.3) | 0.492 | 1.125  (0.450 to 2.813) 0.801 | 0.515  (0.382 to 0.647) 0.829 |  |
| ^$^TJC | 0 | 9  (10)  (0 to 28) | 14  (14.25)  (3 to 28) | 0.019 | 0.937  (0.881 to 0.996)  0.037 | 0.342  (0.219 to 0.464) 0.011 |  |
| ^$^SJC | 0 | 4  (7.75)  (0 to 19) | 4.5  (7)  (0 to 19) | 0.984 | 1.006  (0.921 to 1.100) 0.887 | 0.501  (0.369 to 0.634) 0.984 |  |
| ^$^CRP | 0 | 8.15  (26.9)  (0 to 122) | 9.20  (24.9)  (0.06 to 113) | 0.886 | 0.996  (0.980 to 1.013) 0.669 | 0.510  (0.374 to 0.646) 0.889 |  |
| ^$^GH-VAS | 18 | 34  (42)  (5.0 to 97) | 53  (45)  (14 to 89) | 0.088 | 0.984  (0.964 to 1.005) 0.143 | 0.368  (0.227 to 0.509) 0.067 |  |
| ^$^DAS4CRP | 0 | 4.25  (2.09) 5.92(7.61-1.69) | 5.29  (1.84)  (7.66-2.56) | 0.131 | 0.749  (0.521 to 1.077) 0.119 | 0.398  (0.266 to 0.530) 0.131 |  |
| ^$^Naive T-cells | 0 | -3.80  (25.1)  (-33. to +25) | -3.9  (15.7)  (-30.4 to +36.2) | 0.731 | 1.008  (0.979 to 1.039) 0.577 | 0.523  (0.388 to 0.658) 0.736 |  |
| ^$^IRC | 2 | 4.00  (1.63)  (0.70 to 22.0) | 4.00  (4.93)  (0.9 to 20.00) | 0.940 | 0.958  (0.859 to 1.067) 0.434 | 0.495  (0.354 to 0.636) 0.944 |  |
| ^$^Treg | 2 | -1.43  (2.39)  (-5.02 to 6.26-) | -0.83  (1.85)  (-3.92 to 2.04-) | 0.194 | 0.871  (0.660 to 1.150) 0.331 | 0.411  (0.281 to 0.541) 0.179 |  |

$Numerical variables reported as median (IQR)[min to max]; #Categorical variables reported as number and frequency (%) of patients. HLA (SE), human leucocyte antigen (shared epitope); TJC, tender joint count; RF, rheumatoid factor; Treg, regulatory T-cells; IRC, inflammatory-related cells; ^$^ normalised frequency, OR, odd ratio; AUC area under the roc curve, SEN, sensitivity, SPE, specificity, PPV/NPV, positive / negative predictive value. CI, Confidence interval.

**Supplementary Figure S4**: AUROC graphical representation of the performances of models using Forward logistic regressions comparing model with and without T-cell biomarkers.


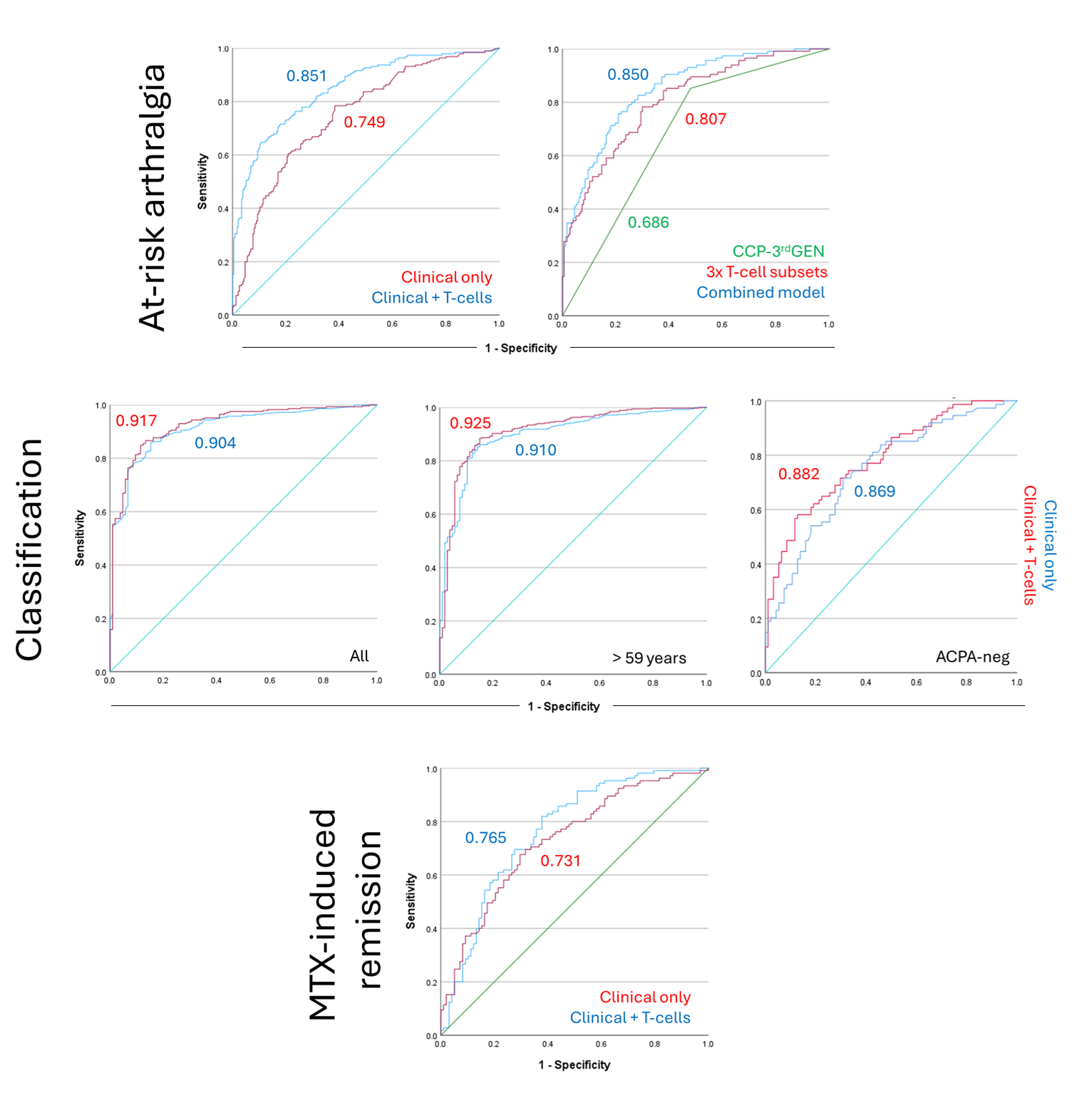


**Discussion of the Biomarker value of the T-cell subset**

In arthralgia/ACPA+ individual, all 3 biomarkers were clearly predictors, both individually and in combination with other variables. Changes from low to high risk over time, is justifying the need to repeat analysis on a yearly basis. Treg appears the most predictive of progression with the best univariate and multivariate prediction indexes. IRC were previously shown to contribute to the prediction of imminent progression (COX-regression ^[2]^). The independent added-value of the T-cell biomarkers, notably over an CCP-research test, was clearly observed for all performances indices (Figure-1).

At the time when IA develops, naïve were still highly associated with RA classification individually and in models. Furthermore, in patients who could not be classified at 1^st^ visit to the EAC (64 progressing over 24 months), modelling T-cells biomarkers showed a gain of +0.070 AUC/+1.7% Sensitivity/+2.4% NPV (data not shown), only selecting ACPA versus ACPA/naive. Treg were no longer strongly associated, after onset of symptoms, and IRC appear to have high specificity for IA (UA, RA, PsA and also other inflammatory conditions) as well as in Chron’s disease as previously observed ^[13]^. Despite this, both Treg/IRC had significant contribution to the combined model. The best added-value of these biomarker for classification was definitely associated with the ACPA-negative patients, with a +13% gain in PPV in a group in clear need for such biomarker.

These are however probably most suited to a population of patients >59 likely due to a possible confounding effect of concomitant ACPA+ OA as previously observed, that we could not address directly (not recorded). Eliminating the age/OA confounding effect in the EAC could also be achieved by using an alternative CCP-test in the >60 years old ACPA+ patients with a high naïve/IRC and low Treg, suspected of having OA and possibly over-diagnosed as RA. Finally, a association between loss of Treg with ACPA+ diseases may be suggested as Treg are not involved in ACPA- RA classification. In contrast, a clear Treg loss is seen in ACPA+ RA (median -1.5%) but not in ACPA+ non-RA (n=10, +0.85%, p=0.008).

Achieving clinical remission, defined by a Disease Activity Score in 28 joints (DAS28) of <2.6, is nowadays the primary goal of therapeutic intervention in early RA ^[14]^ and altogether appear to be achievable, while only in a 45-50% of patients whit ~10% achieve drug free remission ^[15-17]^). Only naïve cells were predictive of MTX-induced remission with added value over clinical data only, but not of TNF-inhibitor-induced remission where no variable currently predict outcome. There was clear increased model performance, allowing for stratification of patients with low-chance of responding to MTX for alternative/more aggressive therapy. Applying a cut-off for dichotomisation of naïve data (>+2.5%) (previously reported at +2% in 70 patients ^[18]^) to this group of 221 patients, 75/116 (65%) patients achieved remission in high-chance group and 57/87 (66%) did not in the low chance group (X^2^ p<2 x10^-5^). Applying this cut-off to the 78 patients receiving TNF-inhibition, 48 had a low-chance of achieving remission on MTX, and 30/48 (62.5%) actually achieved it, at the same rate than those in the high-change group (19/30 (63%)). This provides a strong rational for stratifying early RA patients using naive T-cells, enabling personalisation of the choice of the best drug to achieve remission.

Summary of predictive value of T-cell subsets across the RA continuum.

|  | | univariate analysis | | | multivariate model | | | |  |
| --- | --- | --- | --- | --- | --- | --- | --- | --- | --- |
|  |  | naïve CD4+ | IRC | Treg | naïve CD4+ | IRC | Treg | variables in the model | |
| pre RA | overall progression | Predicitve | predicitve | predicitve | included | included | included | RF, CRP HLA-DR, | |
| RA  diagnostic | ovreall cohort | Predicitve | not | not | included | included | included | age, RF, ACPA, SJC | |
|  | ACPA- patients | Predicitve | predicitve | not | included | included | not | age, RF, SJC | |
|  | >59 years old | Predicitve | predicitve | predicitve | included | included | included | RF, ACPA, SJC | |
|  | Late-onser RA | Not | not | not | not | not | not | ACPA, SJC | |
| 1st line treatment | methotrexate | Predicitve | not | not | included | not | not | smoking, TJC, CRP | |
|  | TNF-inhibitor | Not | not | not | not | not | not | no model computed | |

| Over time | | naïve CD4+ | IRC | Treg |
| --- | --- | --- | --- | --- |
| pre RA | pre-RA duration | **reduces** | **increases** | no change |
| RA diagnostic | symptom duration | no change | no change | no change |
| achieving remission  over 1st treatment | methotrexate | **increases** | no change | no change |
|  | TNF-inhibitor | **increases** | **reduces** | no change |
| over stable  remission | methotrexate | **increases** | no change | no change |
|  | TNF-inhibitor | no change | no change | **increases** |

**Supplementary Figure S5**: Correlations between variables used in the progression and classification models, colours coded for p-values and rho values

Of note, Rho value below -0.600 or above +0.600 are usually considered significant, but none reached this cut-off except for TJC/SJC in early RA.

At-risk/ACPA+ Progressors (n=198) RA classification (n=379)


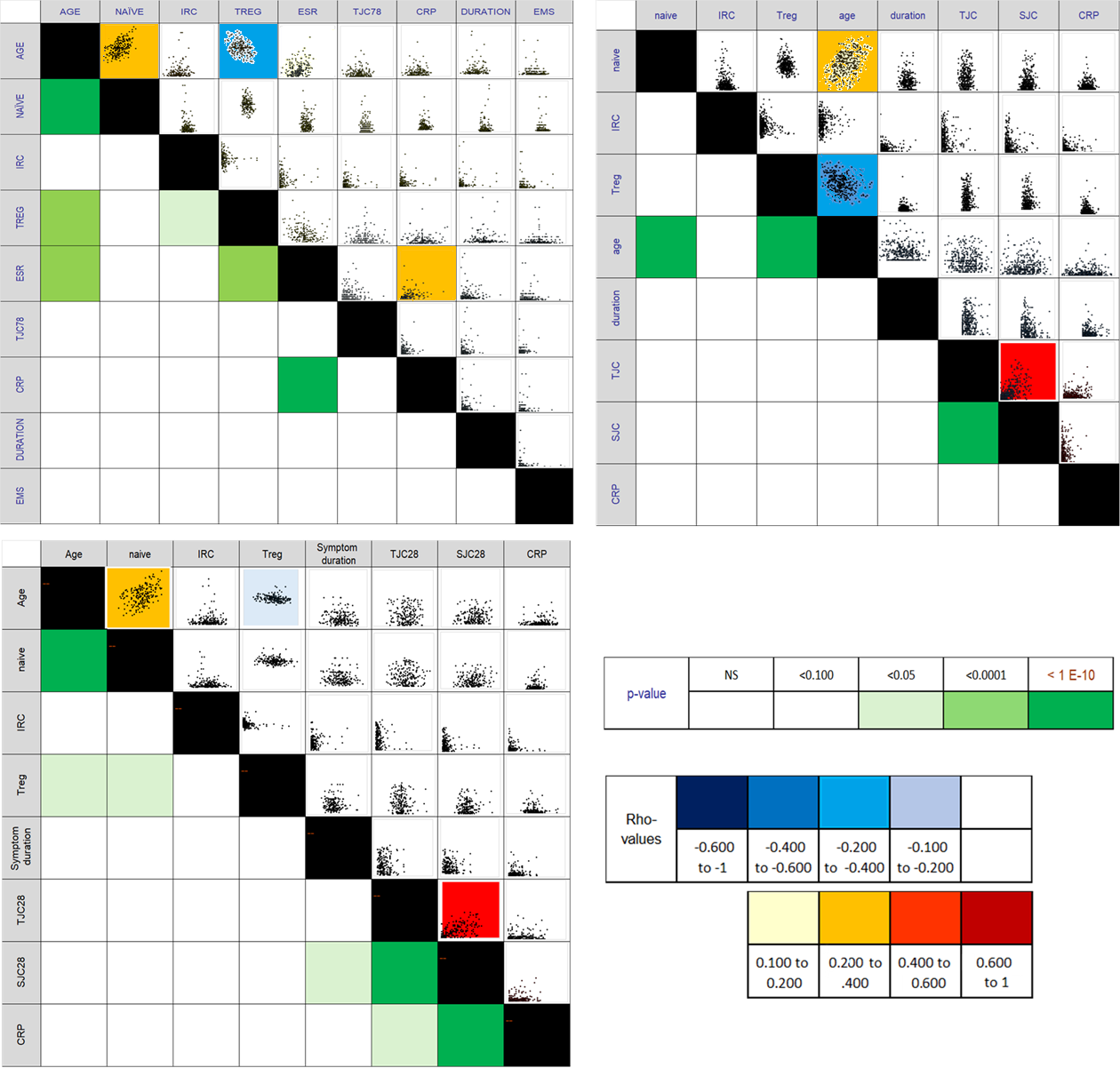


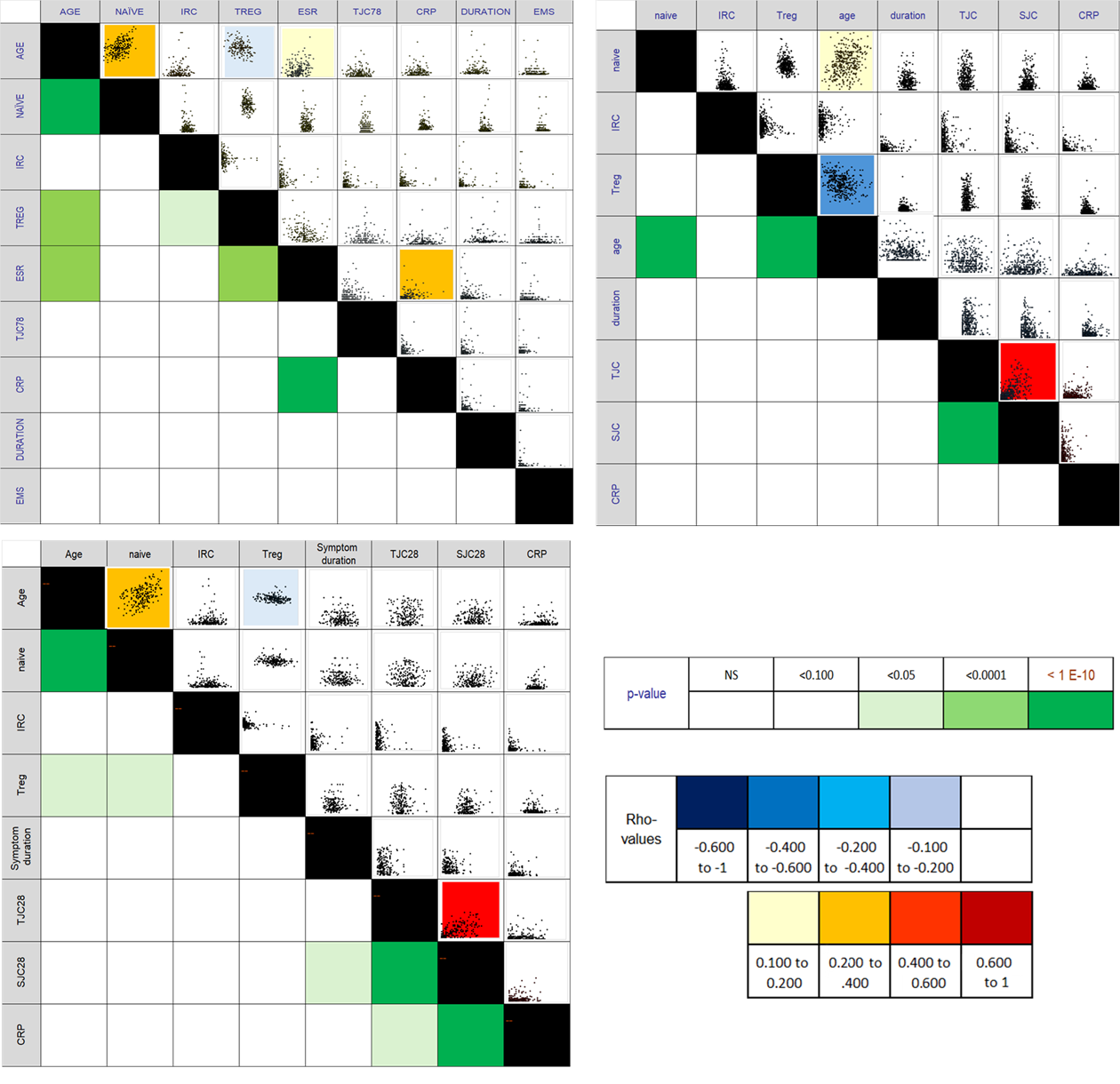


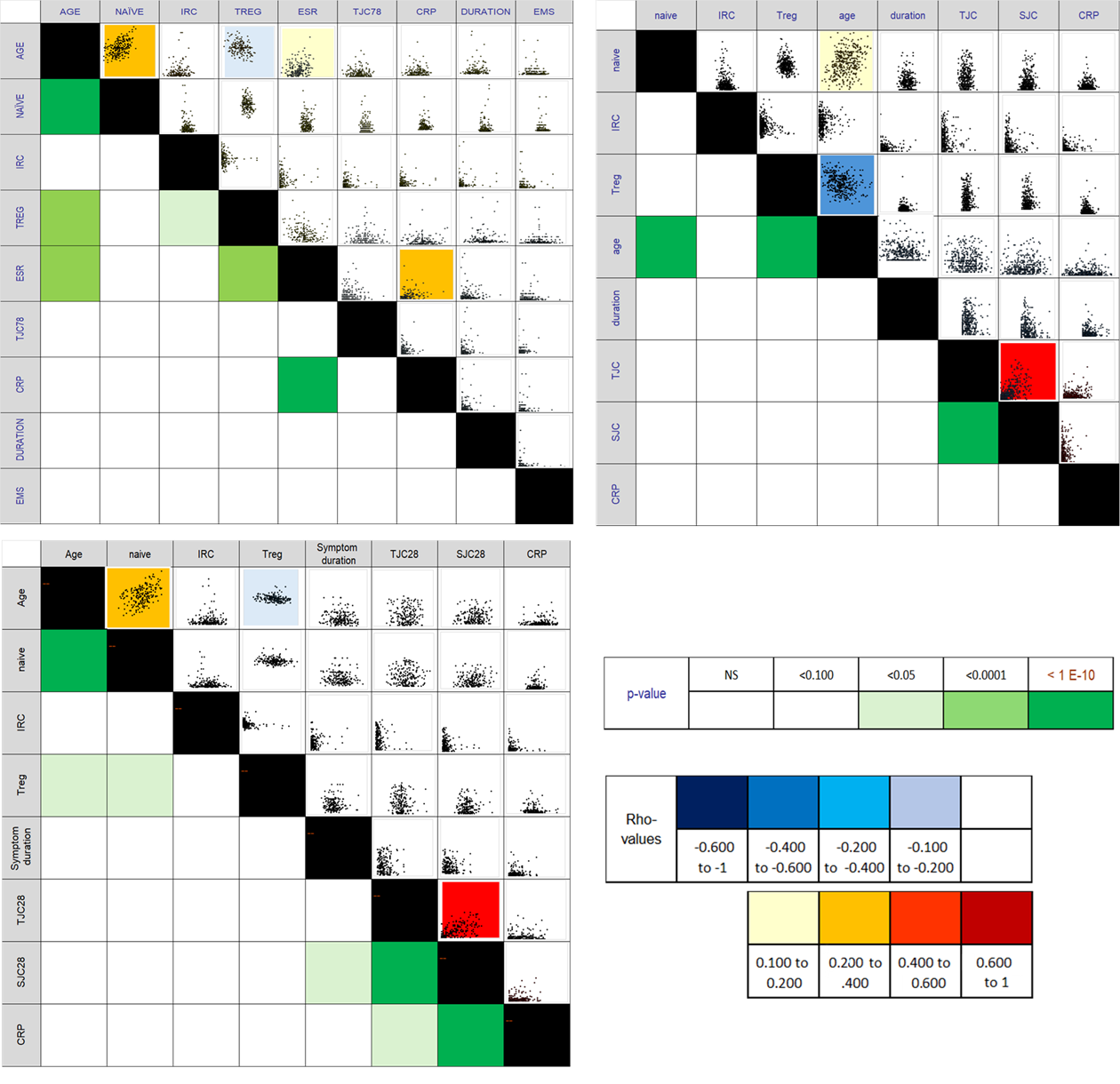


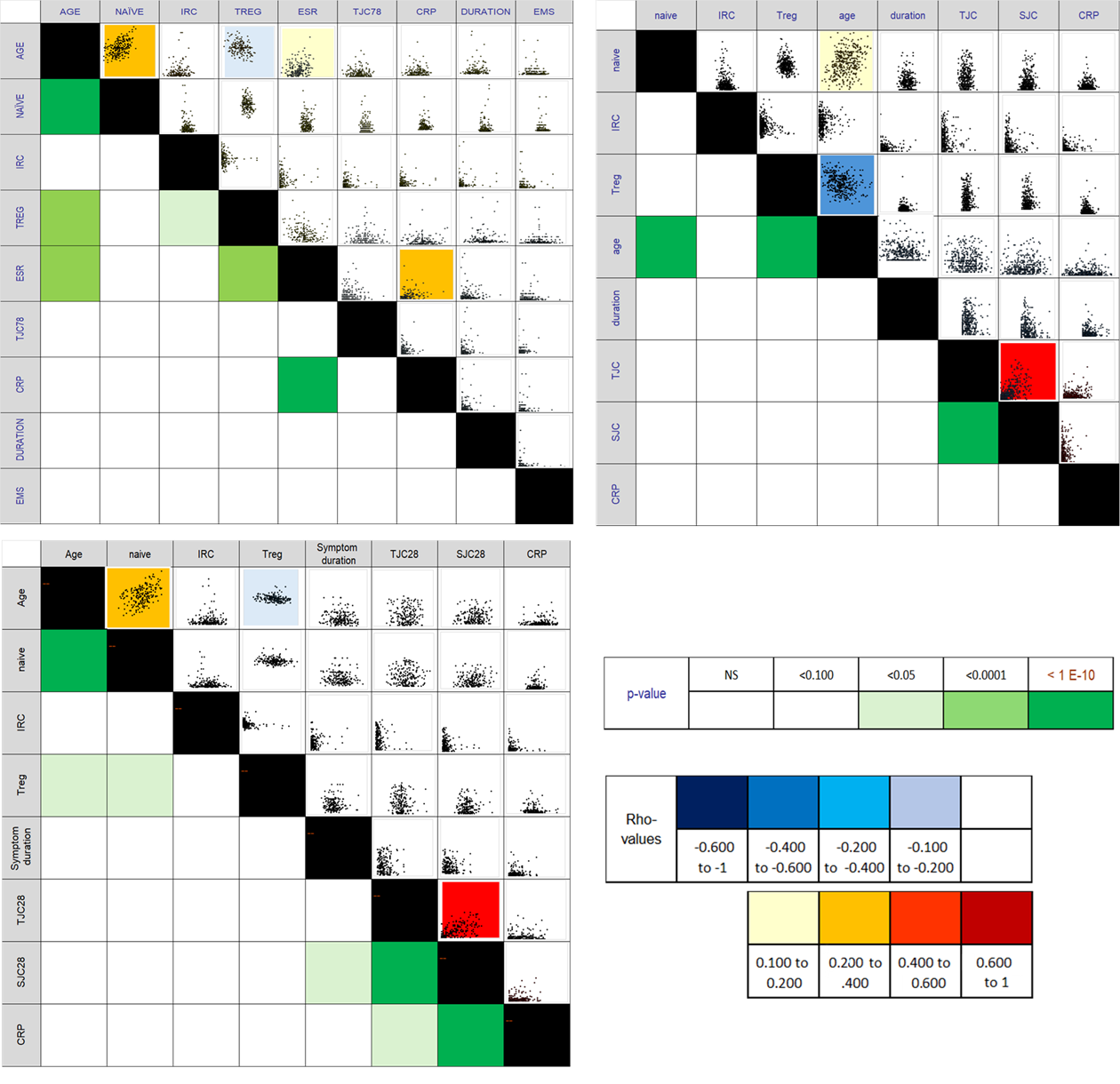


**Antibody pre-coated, dry-tube (DT) validation**

The DT project was driven by the need to improve quality and productivity while reducing the costs/time of the workflow for flow cytometry in health services laboratories. This was a collaboration with Becton Dickinson (BD). The dry-tube (DT) technology is intended to minimise lab procedures (i.e., handling mistake, missing Ab, etc…) and gain time in routine settings as well as to disseminate the test internationally.

Antibody were selected and the DT were manufactured by BD, with a cocktail of 5 antibody dried in a 12 x 75mm bar-coded flow-tube for the T-cell panel for CD4+T subsets and a 4-antibody cocktail for Treg panel. We followed the DT technology protocol for staining (45 minutes for cell surface) and added the intracellular step (FoxP3-Ab) for the Treg panel. Data were acquired and % of subset recorded. Wet tube (WT) classic flow cytometry was performed in parallel. Data were then directly compared. 43 consecutive patients at various stages of the IAC, were included in the study over a period of 3 months. These patients had varied levels of inflammation.

**Staining results**

The marker expression profile of T-cells stained with the DT technology was satisfactory, not compromising the separation of subsets populations compared to WT assay. For the Treg panel, staining was not detectable for FoxP3-Alexa488 in DT. The same antibody used in WT assay was however detecting FoxP3+ cells. The staining for a “putative” Treg gate based only on cell surface proteins (CD4/CD127/CD25) showed similar staining pattern.

**
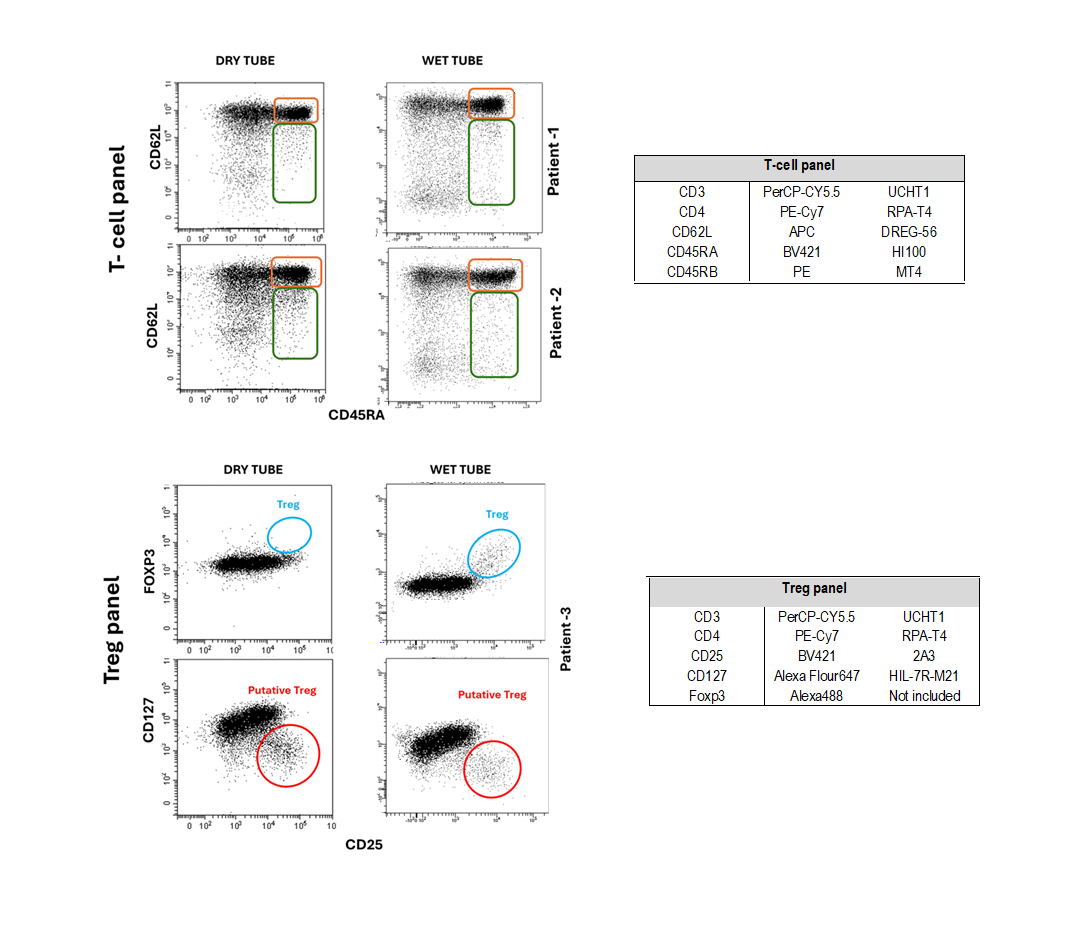
**

Data obtained were then analysed using comparison methods. Data first were described at group levels, showing similar distribution with a small shift toward higher frequencies for IRC using DT (due to better discrimination with the anti-CD45RA antibody). The agreement between the DT / WT was satisfactory for naïve cells (rho=0.930, p<0.0001), while less so for IRC (rho=0.522, p=0.037). Bland-Altman (BA) plots were generated and Intraclass correlation coefficient (ICC) calculated**.** For naïve cells, BA plot showed no evidence of bias. The range in the difference between the 2 techniques was within the range by which measurements differ for naïve CD4+T-cells. For IRC enumeration, the median difference was different from zero, with a pronounced bias at –1.29%. A bias in data distribution pattern was observed with low frequencies data points tending to scatter above the median line, but below at high frequencies. The BA plot suggests a range in measurement difference, with a definite bias (p=0.006) mainly observed for high frequencies (>2.5%) being over-estimated by the DT, while at low frequencies (<2.5%) they were not affected. Reliability remained high over the overall range, despite higher determination in DT with an ICC=0.962. This suggests that IRC results obtained by WT are also reproducible using DT technology.

**Alternative strategy for a Treg phenotype (with no intracellular staining)**

Considering that FoxP3 staining is not compatible with the DT technology, we investigated whether a 2 cell surface marker strategy could be used to replace the 3 markers, which would allow use the DT but also lighten the procedure for a test to be adopted more widely.

We analysed 220 .fcs files (HC, and at-risk participants) for a 2- (cell surface marker) (CD25^high^CD127^low^) versus 3- (cell surface + intracellular) marker (CD25^high^FoxP3+CD127^low^) gating for Treg. We observed high correlation between the 2 datasets (left panel, rho=0.860) suggesting that a 2-marker strategy may be suitable to utilise the Treg biomarker. The 2-marker gating over-estimated Treg compared to 3-marker-gating (average +2.5%). Using 70/215 data point from HC, we re-calculated the regression curve and then normalised data for the 2-marker results.

There was still statistical difference between at-risk Pr (n=70) and NP (n=80) (p=0.00045) although it was not as strong as with the 3-marker strategy (middle panel, p=0.0001). OR/AUC were calculated and showed predictive values again slightly less good (OR=0.721, p=0.003 versus OR=0.630, p<0.0001, and right panel, AUC=0.275 versus AUC=0.393). Risk stratification on 2-marker Treg still suggested a clear association with progression (chi^2^ p=0.021) compared to 3-marker (p=0.00022). Accuracy of the 2-marker Treg risk was 60% and that of the 3-marker was 66%.

In this subgroup of 150 participants, multivariate analysis showed only Treg (both strategies) naïve and IRC with RF and smoking as significantly different between Pr (n=70) and NP (n=80). Logistic binary regression did not allow to include all variables, so we used these 5 variables only in models alternating 2- and 3-marker Treg data. In both models, only Treg and naive contributed significantly (accuracy 70% and 72.3%, AUC=0.718 and 0.768 respectively for 2- and 3-marker). The 3 other parameters (IRC/RF/Smoking) clearly showed increased performance in the 2-marker model when compared to a model using only naïve/Treg (accuracy 64% and AUC=0.697) however, they had no effect when using the 3-marker Treg data (accuracy 72.1%, AUC=0.762) suggesting means of mitigating the loss of predictive value of using 2-marker Treg data.


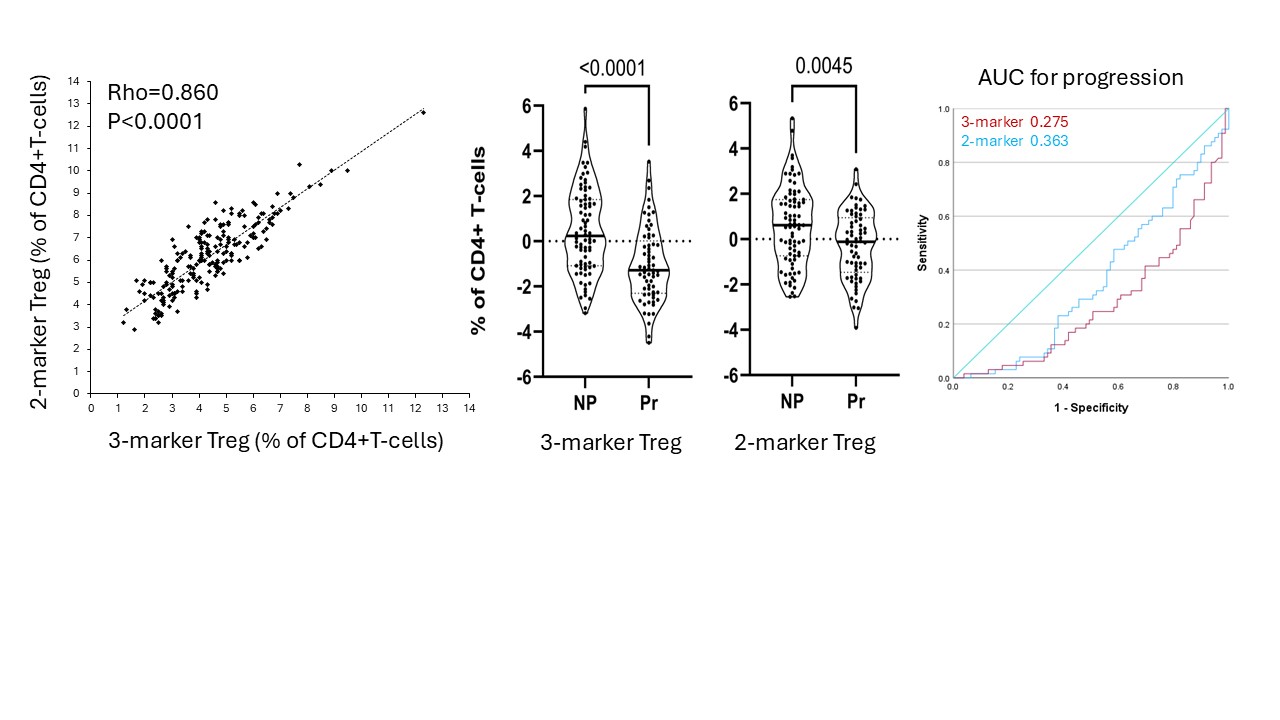


**References**

1. Ponchel, F., A.N. Burska, L. Hunt, H. Gul, T. Rabin, R. Parmar, M.H. Buch, P.G. Conaghan, and P. Emery, *T-cell subset abnormalities predict progression along the Inflammatory Arthritis disease continuum: implications for management.* Scientific Reports, 2020. **10**(1): p. 3669.

2. Anioke, I., L. Duquenne, R. Parmar, K. Mankia, F. Shuweihdi, P. Emery, and F. Ponchel, *Lymphocyte subset phenotyping for the prediction of progression to inflammatory arthritis in anti-citrullinated-peptide antibody-positive at-risk individuals.* Rheumatology, 2023: p. kead466.

3. Rakieh, C., J. Nam, L. Hunt, E. Hensor, S. Das, L. Bissell, E. Villeneuve, D. McGonagle, R. Hodgson, and A.J.A.o.t.r.d. Grainger, *Predicting the development of clinical arthritis in anti-CCP positive individuals with non-specific musculoskeletal symptoms: a prospective observational cohort study.* Annals of the Rheumatic Diseases, 2015. **74**(9): p. 1659-1666.

4. Hunt, L., E. Hensor, J. Nam, A. Burska, R. Parmar, P. Emery, and F. Ponchel, *T cell subsets: an immunological biomarker to predict progression to clinical arthritis in ACPA-positive individuals.* Annals of the Rheumatic Diseases, 2016. **75**(10): p. 1884-1889.

5. Di Matteo, A., K. Mankia, J.L. Nam, E. Cipolletta, L. Garcia-Montoya, L. Duquenne, E. Rowbotham, and P. Emery, *In anti-CCP+ at-risk individuals, radiographic bone erosions are uncommon and are not associated with the development of clinical arthritis.* Rheumatology, 2020. **60**(7): p. 3156-3164.

6. Emery, P., S. Horton, R.B. Dumitru, K. Naraghi, D. van der Heijde, R.J. Wakefield, E.M.A. Hensor, and M.H. Buch, *Pragmatic randomised controlled trial of very early etanercept and MTX versus MTX with delayed etanercept in RA: the VEDERA trial.* Ann Rheum Dis, 2020. **79**(4): p. 464-471.

7. Nam, J.L., E. Villeneuve, E.M. Hensor, R.J. Wakefield, P.G. Conaghan, M.J. Green, A. Gough, M. Quinn, R. Reece, S.R. Cox, M.H. Buch, D.M. van der Heijde, and P. Emery, *A randomised controlled trial of etanercept and methotrexate to induce remission in early inflammatory arthritis: the EMPIRE trial.* Ann Rheum Dis, 2014. **73**(6): p. 1027-1036.

8. Tony, H., P. Roll, H. Mei, E. Blümner, A. Straka, L. Gnuegge, and T. Dörner, *Combination of B cell biomarkers as independent predictors of response in patients with RA treated with rituximab.* Clin Exp Rheumatol, 2015. **33**(6): p. 887-894.

9. Yu, N., X. Li, W. Song, D. Li, D. Yu, X. Zeng, M. Li, X. Leng, and X. Li, *CD4+ CD25+ CD127 low/− T cells: a more specific Treg population in human peripheral blood.* Inflammation, 2012. **35**: p. 1773-1780.

10. Santegoets, S.J., E.M. Dijkgraaf, A. Battaglia, P. Beckhove, C.M. Britten, A. Gallimore, A. Godkin, C. Gouttefangeas, T.D. de Gruijl, and H.J. Koenen, *Monitoring regulatory T cells in clinical samples: consensus on an essential marker set and gating strategy for regulatory T cell analysis by flow cytometry.* Cancer Immunology, Immunotherapy, 2015. **64**: p. 1271-1286.

11. Bartlett, J. and C. Frost, *Reliability, repeatability and reproducibility: analysis of measurement errors in continuous variables.* Ultrasound in Obstetrics and Gynecology: The Official Journal of the International Society of Ultrasound in Obstetrics and Gynecology, 2008. **31**(4): p. 466-475.

12. Koo, T.K. and M.Y. Li, *A guideline of selecting and reporting intraclass correlation coefficients for reliability research.* Journal of chiropractic medicine, 2016. **15**(2): p. 155-163.

13. Burgoyne, C., S. Field, AK Brown, E. Hensor, A. English, S. Bingham, R. Verburg, U. Fearon, C. Lawson, H. PJ, L. Straszynski, D. Veale, P. Conaghan, M. Hull, J. van Laar, A. Tennant, P. Emery, J. Isaacs, and F. Ponchel, *Abnormal T-cell differentiation persists in rheumatoid arthritis patients in clinical remission and predicts relapse.* Ann Rheum Diseases, 2007. **doi:10.1136/ard.2007.073833**

14. Smolen, J.S., F.C. Breedveld, G.R. Burmester, V. Bykerk, M. Dougados, P. Emery, T.K. Kvien, M.V. Navarro-Compán, S. Oliver, M. Schoels, M. Scholte-Voshaar, T. Stamm, M. Stoffer, T. Takeuchi, D. Aletaha, J.L. Andreu, M. Aringer, M. Bergman, N. Betteridge, H. Bijlsma, H. Burkhardt, M. Cardiel, B. Combe, P. Durez, J.E. Fonseca, A. Gibofsky, J.J. Gomez-Reino, W. Graninger, P. Hannonen, B. Haraoui, M. Kouloumas, R. Landewe, E. Martin-Mola, P. Nash, M. Ostergaard, A. Östör, P. Richards, T. Sokka-Isler, C. Thorne, A.G. Tzioufas, R. van Vollenhoven, M. de Wit, and D. van der Heijde, *Treating rheumatoid arthritis to target: 2014 update of the recommendations of an international task force.* Ann Rheum Dis, 2016. **75**(1): p. 3-15.

15. Quinn, M.A. and P. Emery, *Window of opportunity in early rheumatoid arthritis: possibility of altering the disease process with early intervention.* Clin Exp Rheumatol, 2003. **21**(5 Suppl 31): p. S154-157.

16. Kedra, J., A. Lafourcade, B. Combe, M. Dougados, D. Hajage, and B. Fautrel, *Positive impact on 10-year outcome of the window of opportunity for conventional synthetic DMARDs in rheumatoid arthritis: results from the ESPOIR cohort.* RMD open, 2022. **8**(1).

17. van Nies, J.A., R. Tsonaka, C. Gaujoux-Viala, B. Fautrel, and A.H. van der Helm-van Mil, *Evaluating relationships between symptom duration and persistence of rheumatoid arthritis: does a window of opportunity exist? Results on the Leiden early arthritis clinic and ESPOIR cohorts.* Ann Rheum Dis, 2015. **74**(5): p. 806-812.

18. Ponchel, F., A. Burska, N, , L. Hunt, H. Gul, R. T, R. Parmar, M. Buch, H,, P. Conaghan, and P. Emery, *T-cell subset abnormalities predict progression along the Inflammatory Arthritis disease continuum: implications for management.* Nature Scien Reports, 2020. **10**: p. 3669-3678.
